# Plant microbiota controls an alternative root branching regulatory mechanism in plants

**DOI:** 10.1073/pnas.2301054120

**Published:** 2023-04-03

**Authors:** Mathieu Gonin, Isai Salas-González, David Gopaulchan, Juan P. Frene, Stijn Roden, Bram Van de Poel, David E. Salt, Gabriel Castrillo

**Affiliations:** ^a^School of Biosciences, University of Nottingham, LE12 5RD, United Kingdom; ^b^Center for Genomics Sciences, Universidad Nacional Autónoma de México, 04510 Mexico City, Mexico; ^c^Future Food Beacon of Excellence, University of Nottingham, LE12 5RD, United Kingdom; ^d^Division of Crop Biotechnics, Department of Biosystems, KU Leuven, 3001 Leuven, Belgium; ^e^Leuven Plant Institute, KU Leuven, 3001 Leuven, Belgium

**Keywords:** lateral roots, plant–microbe interactions, auxin independent mechanism, ethylene signaling, alternative pathway

## Abstract

The establishment of beneficial interactions with microbes has helped plants to modulate root branching plasticity in response to environmental cues. However, how the plant microbiota harmonizes with plant roots to control their branching is unknown. Here, we show that the plant microbiota influences root branching in the model plant *Arabidopsis thaliana*. We define that the microbiota’s ability to control some stages in root branching can be independent of the phytohormone auxin that directs lateral root development under axenic conditions. In addition, we revealed a microbiota-driven mechanism controlling lateral root development that requires the induction of ethylene response pathways. We show that the microbial effects on root branching can be relevant for plant responses to environmental stresses. Thus, we discovered a microbiota-driven regulatory pathway controlling root branching plasticity that could contribute to plant adaptation to different ecosystems.

## Significance

The mutualistic interactions between plants and microbes have coevolved controlling the development of root branching in land plants. However, the mechanisms of how the plant microbiota contributes to root branching are unknown. Using binary interactions, we show that the plant microbiota regulates root architecture in the model plant *Arabidopsis thaliana*. We establish that the microbiota effect on root branching can be independent of the phytohormone auxin signaling and requires the induction of the phytohormone ethylene. In microbiota reconstitution experiments with synthetic and natural microbial communities, we found that the microbial effect on root branching is important for plant adaptation to abiotic stresses. In natural ecosystems, the microbial control exerted on roots likely contribute to plant adaptation to changing environments.

Since their colonization of land, plants have established beneficial interactions with a myriad of plant-colonizing microorganisms, called the plant microbiota (1, 2). These interactions are critical for plant survival under conditions of reduced nutrient and water availability (1, 3–6). Among other mechanisms, microbial activity is known to support the functions of roots, below-ground structures of vascular plants, such as anchoring and uptake of water and mineral nutrients (6, 7). This microbial capability is linked to the microbiota’s capacity to induce changes in root branching. Arbuscular mycorrhizal fungi increase lateral root development in monocotyledons and dicotyledons plants (8), and nitrogen-fixing bacteria induce the formation of root nodules via recruitment of lateral root developmental components (9). Examples of commensal members of the plant microbiota with a positive effect on root branching have been also described (10, 11).

In *Arabidopsis thaliana* (common name *Arabidopsis*), lateral roots are formed from root pericycle founder cells, which after identity acquisition, start a process of cell divisions leading to the formation of lateral root primordia. Additional divisions produce a dome-shaped structure that extends through the external root tissues until emergence (12). Under axenic conditions, in the absence of microbiota, the different steps of root branching are controlled by the phytohormone auxin (13, 14). Other processes, linked to auxin signaling, such as intercellular adhesions (15), the deposition of the aliphatic polyester suberin (16), or changes in the phytohormones cytokinin (17) and ethylene (18) in the root have been found to be relevant for the initiation and emergence of the lateral root primordium in axenic roots. This auxin regulatory network also controls aspects of the root’s interaction with beneficial microbiota (7). However, roots of early land plants appear to branch through bifurcation of the root tip in an auxin-independent process (19). It is therefore likely that plants and microbes coevolved a mechanism of root branching that can be independent of auxin. This auxin-independent ancestral coordination between plants and microbes may have been partially conserved in more recently evolved land plants, influencing lateral root formation, plant performance, and adaptation to different soils.

Here, we have characterized the microbiota’s effect on plant root architecture, revealing that the plant microbiota largely controls features of root development, especially those related to root branching. Colonization experiments using the basal plant *Selaginella moellendorffii* and *Arabidopsis* mutants, impaired in lateral root development, demonstrated that the microbiota controls stages of lateral roots independently of auxin signaling. Using an auxin biosynthesis inhibitor and an auxin biosensor, we show that auxin levels in the root do not change in response to microbial colonization, indicating that the microbial effect on lateral root density does not require the induction of auxin biosynthesis. Transcriptomic analysis of wild-type *Arabidopsis* plants and lateral root mutants point to the induction of ethylene response as positively affecting lateral root development. Furthermore, characterization of lateral root development in ethylene mutants in response to the microbiota revealed an alternative regulatory pathway controlling the coordination between the lateral root endogenous developmental program and the resident microbiota. We reason that the microbiota-driven effect on lateral roots contributes to the plasticity of root architecture, which is critical for plant survival in changing ecosystems.

## Results

### Plant Microbiota Alters Root Branching.

**Fig. 1.**
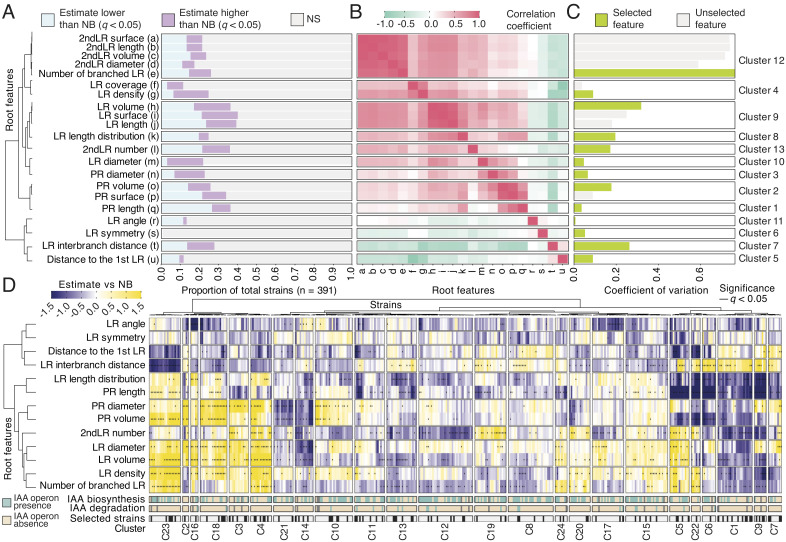
Bacterial isolates influence root architecture. (*A*) Bar graph showing the bacterial strains’ effects on root architecture features. In the graph, different colors represent the proportion of bacterial strains, out of a total of 391, able to induce a positive (purple) or a negative (blue) effect on individual root features. (*B*) Heatmap showing the Pearson’s correlation coefficient from each pairwise comparison between the root architecture features quantified in response to the individual bacterial isolate collection. See *Materials and Methods* section for the root architecture feature definitions. (*C*) Bar-graph showing the coefficient of variation of each root feature in response to individual bacterial strains. In the figure, panels *A*, *B*, and *C* have been hierarchically clustered according to the correlation coefficients from panel *B* to define clusters of highly correlated root features. Within each cluster, the feature with the highest coefficient of variation was selected as a root architecture marker (green bars in panel *C*). (*D*) Heatmap showing the bacterial strains’ estimated effect on selected root architecture features with respect to the uninoculated control. Values that are significantly different from the uninoculated control (Wilcoxon signed-ranked test, *q* value < 0.05) are highlighted with a small vertical line. The values have been clustered according to the bacterial treatment and their effect on the root architecture feature quantification. Colors on the horizontal bars in the *Bottom* of the panel, from *Top* to the *Bottom*, represent the presence of known auxin (IAA) biosynthetic or degrading operons in the available bacterial genomes and the bacterial strains selected for further experiments, respectively. For this experiment, we used at least two independent biological replicates per bacterial condition with 10 plants each.

To systematically deconstruct the plant microbiota’s influence on root morphological traits, we exposed wild-type *Arabidopsis* (accession Col-0) plants to 391 individual bacterial strains (*SI Appendix*, Fig. S1*A* and Dataset S1) that recapitulate the bacterial phylogenetic diversity found in healthy *Arabidopsis* plants grown in natural soils (5, 6). We systematically screened the bacterial collection in monoassociation with the plant, in an agar-based medium with replete nutrients, and quantified changes in root architecture induced by individual bacterial isolates (*SI Appendix*, Fig. S1*B*). For each plant, we quantified 21 root features that maximized phenotypic variability in root development in response to individual bacteria (Fig. 1*A*). We found that 89% of the bacterial strains significantly altered at least one feature of the root architecture (Fig. 1*A* and *SI Appendix*, Fig. S1*C*). To remove possible duplicities in the root features analyzed, we clustered a pairwise correlation matrix to identified groups of highly correlated features (Fig. 1*B* and *SI Appendix*, Fig. S1*D*). Within each cluster, the feature showing the highest coefficient of variation in response to bacteria was considered a root architecture marker (Fig. 1*C*). These selected features were used to define 24 different clusters that represent distinct root architecture responses to bacteria (Fig. 1*D* and Dataset S2). For example, in response to bacterial isolates from clusters C3, C4, C16, C18, and C23, plants increased primary and lateral root parameters with a detrimental effect on the distance between lateral roots and in response to isolates from cluster C1, plants reduced the number of lateral roots and the length of the primary root while increasing the separation between lateral roots, and bacteria from cluster C19 promoted the production of secondary lateral roots (Fig. 1*D*, *SI Appendix*, Fig. S1*E*, and Dataset S2). Consistent with previous findings (6, 20), bacterial phylogeny explained most of the root architecture differences across isolates (*SI Appendix*, Fig. S1*F*). We noticed that in clusters C1 and C23, changes in lateral root features coincided with those observed for primary root phenotypes (Fig. 1*D*). We therefore explored whether these two features are associated. We found a weak correlation (R = 0.29, *P* = 4.1 × 10^−9^) between alterations to lateral and primary roots across plants inoculated with the different bacterial strains (*SI Appendix*, Fig. S1*G*). Thus, the variation found in primary root length does not fully explain the variation observed in lateral root density in response to the presence of bacteria (*SI Appendix*, Fig. S1*H*). These results indicate that individual bacteria can modify lateral root formation independently of primary root development. Furthermore, among the features evaluated, lateral-root-related traits showed the highest coefficient of variation (Fig. 1*C*) and the number of bacteria that altered these features was highest across all phenotypes analyzed (Fig. 1 *A* and *B*). This indicates that lateral root development is an important bacterial target to induce modifications in plant root architecture. Therefore, we focused on the feature lateral root density as a proxy in order to comprehensively characterize the effect of the microbiota on lateral root formation.

In *Arabidopsis*, lateral root development is controlled by the plant hormone auxin in axenic conditions (13, 14). Thus, we explored whether the bacterial capacity to induce changes in lateral roots was exclusively linked to the presence of known auxin biosynthetic or auxin-degrading operons in the bacterial genomes (Fig. 1*D*, *SI Appendix*, Fig. S1 *A* and *F*, and Dataset S1). We did not observe significant differences in the distribution of the magnitude of any of the bacterial effects on root morphology between bacteria bearing or lacking known auxin-related operons (*SI Appendix*, Fig. S2*A*). In this line, we did not observe significant differences (PERMANOVA) of the effect of these two groups of bacteria on the different root architecture features within individual clusters from Fig. 1*D* (*SI Appendix*, Fig. S2*B*). Although we cannot rule out the presence of undiscovered auxin-related operons in bacteria lacking known auxin-related operons, this analysis could suggest that members of the plant microbiota might modify aspects of root development independently of their ability to modulate root auxin homeostasis.

### Microbiota Influences Auxin-Independent Root Branching.

**Fig. 2.**
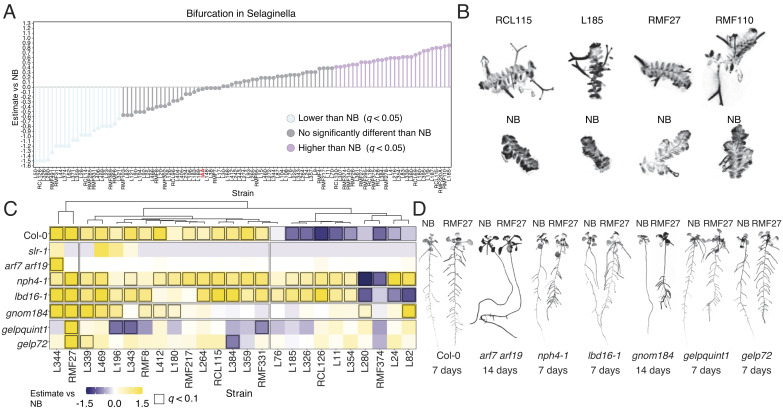
Microbiota control over some stages of lateral root development can be independent of the auxin signaling network. (*A*) Lollipop plots showing the bacterial effect on *Selaginella* branching. The effect of individual bacteria on *Selaginella* root bifurcation was estimated as compared with the uninoculated control. Values colored in purple and blue are significantly higher or lower from the no bacteria control (Dunnet test, *q* < 0.05), respectively. Highlighted in red is auxin-treated *Selaginella* (IAA) that we used as a control. For this experiment we used two independent biological replicates per bacterial condition with 12 explants each. (*B*). Exemplary images of root branching in *Selaginella* explants grown in axenic MS plates (No bacteria, NB) or MS plates inoculated with the bacteria RCL115, L185, RMF27, and RMF110 that induced root bifurcations. (*C*) Heatmap showing the bacterial strains’ estimated effect on lateral root density with respect to the no bacteria control in wild-type Col-0 plants and lateral root mutants *iaa14 slr-1*, *arf7 arf19*, *nph4-1*, *lbd16-1, gnom184*, *gelpquint1*, and *gelp72*, after 7 d of colonization with different bacterial isolates. The values have been clustered according to the bacterial treatments. Values that are significantly different from the uninoculated control are highlighted with a black square (Dunnet test, *q*-value < 0.1). (*D*) Exemplary images of wild-type Col-0 plants and lateral root mutants *arf7 arf19*, *nph4-1*, *lbd16-1, gnom184*, *gelpquint1*, and *gelp72* grown in axenic MS plates (No bacteria, NB) or MS plates inoculated with the bacterium RMF27 that increases lateral root density. To highlight the effect of the bacterium on lateral root formation, some mutants were imaged at 7 d and others at 14 d after inoculation. For this experiment at least two biological independent replicates were used per bacterial treatment with 10 plants each.

To investigate whether the capacity of the microbiota to control aspects of root architecture is retained in plants with auxin-independent mechanisms of branching, we used a representative subset (n = 99) of bacteria that spans across all clusters identified in Fig. 1*D*. This subset included isolates with or without known operons related to auxin synthesis or degradation (Fig. 1*D*, *SI Appendix*, Fig. S1*A*, and Dataset S1). We grew the basal land plant *S. moellendorffii* (*Selaginella*) axenically or in monoassociations with the selected strains under full nutrient conditions. *Selaginella* roots branch via bifurcation, and this process is not responsive to auxin (19). We counted the number of bifurcation events in *Selaginella* roots in response to the various bacterial strains. We observed that 23% and 14% of bacteria tested significantly induced or repressed branching in *Selaginella* roots, respectively (Fig. 2 *A* and *B*). As in *Arabidopsis* (*SI Appendix*, Fig. S2 *A* and *B*), the ability of these bacterial strains to potentially produce or degrade auxin might be independent of its effect on root branching (*SI Appendix*, Fig. S3*A*). These findings indicate that members of the plant microbiota can coordinate with an auxin-independent regulatory mechanism to control root branching in a basal land plant.

To further delineate mechanisms of coordination between lateral root development and the plant microbiota that can be auxin-independent, we selected a tractable subset of 21 bacterial isolates lacking known auxin-related operon in their genomes (Fig. 1*D*, *SI Appendix*, Fig. S1 *A* and *F*, and Dataset S1) that represent different effect intensities on *Arabidopsis* and *Selaginella* root branching (Figs. 1*D* and 2*A*). The use of bacteria lacking known auxin-related operons minimizes their capacity to interfere with the endogenous plant auxin metabolism controlling lateral root development in *Arabidopsis*. We confirmed that 15 of the selected strains were not able to produce or degrade auxin in in vitro assays (*SI Appendix*, Fig. S3*B*). As controls, we included four bacterial isolates having auxin biosynthetic operons in their genomes (*SI Appendix*, Fig. S3*B* and Dataset S1).

We inoculated seedlings of wild-type *Arabidopsis* plants and a selection of seven lateral roots mutants, *iaa14 slr-1*, *arf7 arf19*, *nph4-1*, *lbd16-1, gnom184*, *gelpquint1*, and *gelp72*, that represent different levels of impairment in lateral root development signaling mediated by the phytohormone auxin (13–16) (*SI Appendix*, Fig. S3*C*). SLR/IAA14–ARF7–ARF19 module controls the starting process in lateral root founder cells. The LBD16, a transcription factor, regulates cell cycle genes involved in the formation of the lateral root primordia (13, 14). The ADP ribosylation factor guanine nucleotide exchange factor (ARF-GEF) GNOM controls the balance between esterified and deesterified pectin, a cell wall component, required for proper initiation of lateral root primordia (15). Auxin-induced GDSL-motif–containing enzymes (for example, GELP72) regulate suberin deposition involved in lateral root emergence (16). Under the full nutrient conditions used, most of these mutants showed greater primary root elongation compared to wild-type plants (*SI Appendix*, Fig. S3*D*). We did not observe a significant bacterial effect on lateral root development in the mutant of *IAA14-SLR* that is required for lateral root initiation, a process that is strictly regulated by auxin in *Arabidopsis* (13–16) (Fig. 2*C* and *SI Appendix*, Fig. S3 *E* and *F*). We confirmed, in the rest of the mutants used, a bacterial effect on lateral root density regardless of their level of impairment in the lateral root signaling pathway (Fig. 2 *C* and *D* and *SI Appendix*, Fig. S3 *E* and *F*). We observed that not all bacteria have the ability to induce changes in all mutants tested, but strains such as L344 and RMF27 enhanced lateral root development in the more extreme mutants *arf7 arf19* and *gnom184* (Fig. 2 *C* and *D* and *SI Appendix*, Fig. S3 *E* and *F*). Thus, we demonstrated that control of the microbiota over some stages of lateral root development can be independent of the canonical auxin signaling network that controls lateral root formation in *Arabidopsis* under axenic conditions.

Next, we discarded that the bacterial effect on lateral roots was an indirect effect due to activation of lateral root formation in response to nutrient deficiencies provoked by plant–bacterium competition. Wild-type and mutant plants grown axenically, with no microbiota present, under serial dilutions of nutrient media, which replicated or even reduced plant nutritional status in the presence of the different bacteria (*SI Appendix*, Fig. S4 *A* and *B*), did not induce lateral root formation in either the wild-type plants or the *Arabidopsis* mutants analyzed (*SI Appendix*, Fig. S4 *C* and *D*). Also, we observed no correlation between the bacterial capacity to induce changes in endodermal suberization, important for lateral root emergence (6, 16) and nutrient homeostasis (6), and the lateral root phenotypes in wild-type plants (*SI Appendix*, Fig. S4*E*). Indeed, the bacterium RMF27, which induced the highest lateral root density in Col-0 plants (*SI Appendix*, Fig. S3*E*), also colonized and induced a similar number of lateral roots in the *pCASP1::CDEF1* line expressing the cuticle destructing factor1 that degrades suberin (21) (*SI Appendix*, Fig. S4*F*). All these results further indicate the existence of an alternative mechanism controlling the microbiota’s effect on lateral root development in plants that is independent of the nutritional homeostatic mechanisms influencing lateral root development.

**Fig. 3.**
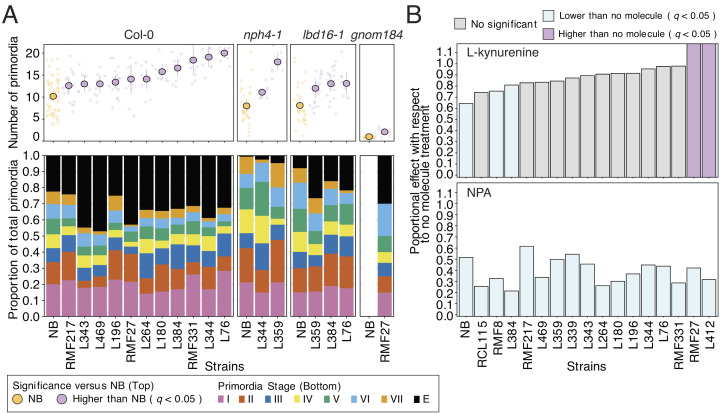
The microbiota positive effect on lateral root development and function can be independent of auxin biosynthesis. (*A*) Stacked bar charts showing the proportion of lateral root primordia at different developmental stages in wild-type plants and lateral root mutants *nph4-1*, *lbd16-1,* and *gnom184* inoculated with a collection of bacterial isolates. Different colors in the chart represent the different lateral root primordia stages [I, II, III, IV, V, VI, VII, and emerged (E)] (22). On top of the figure are pointrange plots showing the total number of lateral root primordia across the different genotypes and bacterial treatments used. In each case, the total number of lateral root primordia in the uninoculated control is colored in yellow and strains that significantly increase the number of primordia with respect to the uninoculated control are colored in purple (Dunnet test, *q* < 0.05). For this experiment, we used 10 plants per bacterial and genotypes condition. (*B*) Bar graph showing the proportional effect of L-Kynurenine (*Top*) and NPA (*Bottom*) on lateral root density as compared to untreated plants (no molecule) in wild-type plants colonized or not with individual bacterial isolates. Different colors represent the significance of the lateral root density change caused by the auxin inhibitors (molecules) as compared with untreated plants. Bars colored in gray denote plants that did not significantly respond to the auxin inhibitor treatments, in purple and blue are plants that increase or decrease the lateral root density in response to the auxin inhibitors, respectively (Dunnet test, *q* < 0.05). We used two independent biological replicates per condition with at least 10 plants each.

To place the effect of the microbiota on the lateral root developmental pathway, we quantified the number of primordia across the different stages of lateral root development in *Arabidopsis* wild-type plants (22) and lateral root mutants in response to a reduced selection of 11 bacteria with a positive effect on lateral root density (Fig. 2 *C* and *D* and *SI Appendix*, Fig. S3 *E* and *F*). We found that in general, the bacterial isolates used increased the number of primordia in wild-type plants and mutants as compared to the uninoculated controls (Fig. 3*A*). We confirmed the results found in wild-type plants (Fig. 3*A*) using the *pSKP2B::GUS* line (23) and *pLBD16:GFP* (24), marker lines for primordia formation (*SI Appendix*, Fig. S5 *A*–*C*). We found that the number of primordia in wild-type plants correlates with the number of emerged lateral roots in response to the bacterial isolates (*SI Appendix*, Fig. S5*D*). This correlation was evident in the case of primordia at stages I, II, and III, before activation of the apical meristem, with its capacity to produce auxin, in newly formed lateral roots (*SI Appendix*, Fig. S5*D*). This indicates that bacterial strains can promote the development of the lateral root primordium once formed likely via the activation of the transition from a quiescent to an active primordium.

Also, we analyzed the bacterial effect on prebranch site formation in the root, a process that determines the spatiotemporal distribution of lateral roots along the primary root (14). We exposed the prebranch site marker line *DR5:Luciferase* to 16 of the 25 initial subsets of individual bacteria lacking auxin operons and known to modify the number of lateral roots (Fig. 2 *C* and *D* and *SI Appendix*, Fig. S3*E*), and we quantified the density of prebranch sites on the primary root axis in all cases. We found that in 81% (13 bacteria) of the cases, bacterial treatments did not modify the density of prebranch sites in the roots (*SI Appendix*, Fig. S5*E*). This result confirmed our hypothesis that, rather than increasing the number of prebranch sites in the root, the microbiota, in general, increases the number of early-stage primordia capable of developing into a lateral root.

Next, we demonstrated using the auxin sensor *DII:VENUS* (25) that the observed increased number of primordia and emerged lateral roots caused by bacteria were not due to changes in auxin signaling in the root (*SI Appendix*, Fig. S6 *A* and *B*). We also found that, in general, wild-type plants inoculated with individual bacterial strains and treated with L-kynurenine (L-Kyn), an auxin biosynthesis inhibitors (26), did not show a significant reduction in lateral root density compared to untreated plants (Fig. 3*B* and *SI Appendix*, Fig. S6*C*). As expected, bacteria-inoculated plants treated with N-1-naphthylphthalamic acid (NPA) that inhibits auxin polar transport (27) necessary for lateral root founder cell priming reduced the lateral root density to the level found in treated-wild-type plants grown under axenic conditions (Fig. 3*B* and *SI Appendix*, Fig. S6*C*). We verified the lack of capacity of the selected bacteria to use NPA and L-Kyn as carbon sources (*SI Appendix*, Fig. S6*D*). Thus, all of these results established that once the prebranch sites have been formed, the root microbiota can control lateral root primordia progression through a mechanism that can be independent of auxin biosynthesis.

### Ethylene Influences Lateral Root Formation.

To identify the alternative mechanism of how the microbiota induces lateral root primordia progression independently of root auxin levels, we performed a transcriptional analysis using roots of wild-type plants and lateral root mutants *arf7 arf19*, *nph4-1*, *lbd16-1*, and *gnom184,* in monoassociation with the 16 bacteria able to restore the lateral root formation in the mutants used (Fig. 2 *C* and *D* and *SI Appendix*, Fig. S3 *E* and *F*). To reduce potential masking effects of auxin signaling, we harvested roots at 7 d after transferring to the bacteria treatment, out of the peak of auxin response activation that in our experimental setup occurred at 4 d post transferring (*SI Appendix*, Fig. S7*A*).

**Fig. 4.**
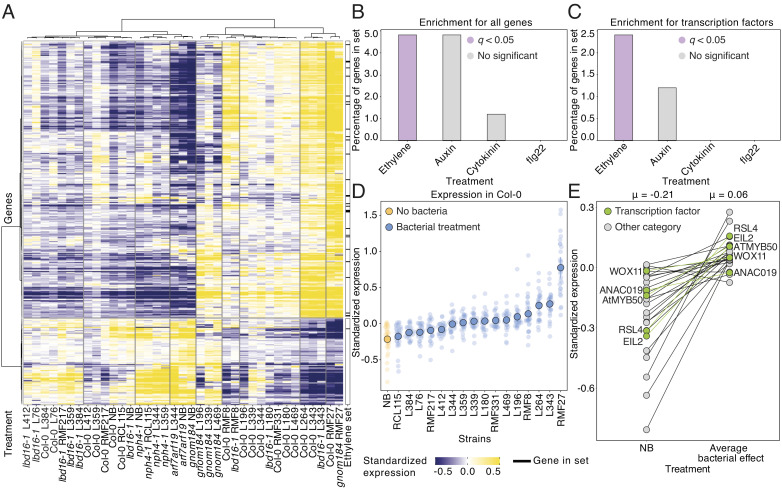
Plant microbiota induces a transcriptional response to ethylene during lateral root formation. (*A*) Heatmap of the 437 differentially expressed genes selected from an RNA sequencing experiment using a hierarchical gene filtering strategy (*SI Appendix*, Fig. S7 *B* and *C*) on transcriptional data of Col-0, the lateral root mutants *arf7 arf19*, *nph4-1*, *lbd16-1*, and *gnom184*, and the plant lateral root phenotypes in response or not (NB) to bacterial isolates. Clusters in the *x* axis are labeled with the plant genotype and the bacterial treatments. Vertical bar on the right shows the prevalence of ethylene core genes identified from the literature (6). (*B*) Bar graphs showing the percentage of genes and (*C*). Transcription factors, from phytohormones (Ethylene, Auxin, Cytokinin) and the pathogen-associated molecular pattern flg22 core genes sets identified from the literature (5, 28–33), found among the high expressed genes (>= 3× log_2_fold change) identified in *A*. Purple color indicates statistical significance (hypergeometric test, *q* < 0.05). See also *SI Appendix*, Fig. S8*A*. (*D*) Standardized expression of the identified 28 ethylene-responsive genes induced in *A*. in axenic plants (NB) or in response to the individual bacteria or (*E*). an average of bacterial treatments. Green color represents transcription factors. The line connecting both points is the difference between uninoculated and bacterial treatments. The mean expression (μ) for each treatment is on the *Top* of the panel. For the RNAseq experiment, we used three independent biological replicates per condition with at least 10 roots each. We repeated this experiment twice (n = 6).

We compared differentially expressed genes in roots of wild-type plants grown axenically with those exposed to each individual bacterium to identify 3,051 genes differentially expressed in wild-type plants in response to the bacterial treatments (*SI Appendix*, Fig. S7 *B* and *C*). We individually filtered our gene selections by comparing them with those genes found significantly expressed in response to individual bacterial strains in the corresponding lateral root mutants *arf7 arf19*, *nph4-1*, *lbd16-1*, and *gnom184* (*SI Appendix*, Fig. S7 *B*–*D*). Finally, the resulting gene sets of 2,122 genes were subdivided by correlating the gene expression with lateral root density measurements found in response to individual bacterial strains (Fig. 4*A* and *SI Appendix*, Fig. S7 *B*–*E*). This hierarchical gene filtering strategy, that uses mutants impaired in the canonical auxin signaling controlling lateral roots and the plant phenotypes, allowed us to identify differentially expressed genes likely involved in the control of stages of lateral root formation independent of these auxin signaling components. Indeed, we found in our final gene selection a significant depletion in unspecific defense-related genes with no correlation to lateral root density, and a high representation of root development genes highly correlated with the lateral root phenotype analyzed (Fig. 4*A* and *SI Appendix*, Fig. S7 *D*–*F*).

Hierarchical clustering analysis of the 437 filtered genes identified two major clusters of differentially expressed genes that responded to the presence of individual bacteria in both wild-type and lateral root mutants (Fig. 4*A*). In line with the bacterial effect on lateral root formation, these clusters were enriched in induced genes related to root development and in repressed genes involved in defense (*SI Appendix*, Fig. S7*F*).

We noticed that genes responsive to ethylene (6), a plant hormone known to be involved in lateral root development in axenic plants (18), were overrepresented in our final selection of genes (Fig. 4 *A* and *B* and *SI Appendix*, Fig. S8 *A* and *B*). The enrichment in ethylene-related genes was more evident in highly expressed genes, more than 3-log_2_-fold change, that were also significantly enriched in transcription factors related to ethylene signaling (Fig. 4 *A*–*C*, *SI Appendix*, Fig. S8 *A* and *B*, and Dataset S3). We did not find these levels of enrichments for other phytohormones such as auxin and cytokinin or the bacterial pathogen-associated molecular pattern (PAMP) flg22, present in our bacterial collection, signals known to influence lateral root formation (Fig. 4 *B* and *C*, *SI Appendix*, Fig. S8*A*, and Datasets S3 and S4). Furthermore, we found that this ethylene gene set was highly induced in both wild-type plants and lateral root mutants, in response to bacterial isolates bearing or not known ethylene biosynthetic operons in their genomes (Fig. 4 *A*, *D*, and *E*, *SI Appendix*, Fig. S8*C*, and Dataset S4). We noticed that half of these genes are coexpressed with genes important for root function (*SI Appendix*, Fig. S8*D*). Thus, we hypothesized that an alternative pathway, involving the induction of ethylene responses in the root, might control stages of lateral root development in response to the plant microbiota.

**Fig. 5.**
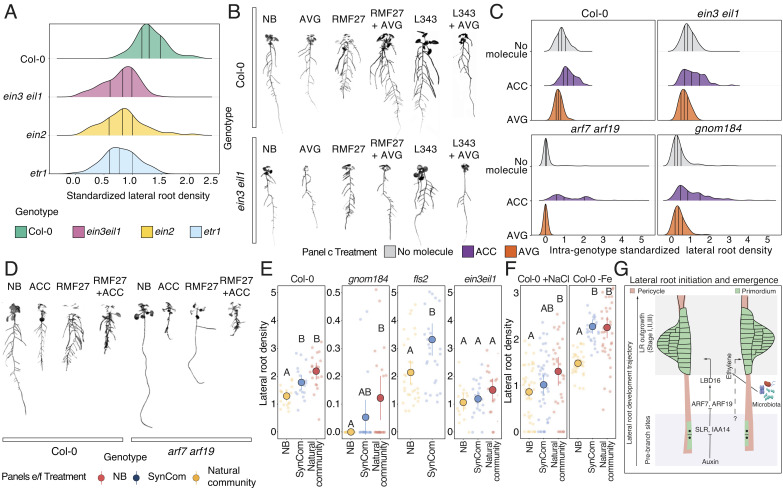
Plant microbiota controls some stages of lateral root development thorough the induction of ethylene response in the plant. (*A*) Distribution of the bacterial magnitude effect on lateral root density in wild-type plants Col-0 and the ethylene mutants *ein3 eil1*, *ein2*, and *etr1* colonized with individual bacterial strains. We used two independent biological replicates per condition with 10 plants each, and we repeated this experiment twice. (*B*) Exemplary images showing the effect of the ethylene inhibitor AVG on lateral root density in wild-type plants and ethylene mutant *ein3 eil1* colonized or not with bacterial isolates. (*C*) Distribution of the bacterial magnitude effect on lateral root density in wild-type plants Col-0, the ethylene mutants *ein3 eil1*, and the lateral root mutants *arf7 arf19* and *gnom184* colonized only with individual bacterial strains (no molecule) or treated with the ethylene precursor ACC or with the ethylene biosynthesis inhibitor AVG. Within each genotype, we standardized the bacterial effects across treatments to visualize the effect of ACC and AVG. We used two independent biological replicates per condition with 10 plants each. (*D*) The images show examples of wild-type plants and the lateral root mutant *arf7 arf19* grown under axenic conditions and treated or not with the ethylene precursor ACC or colonized by the bacterium RMF27 and treated or not with ACC. (*E*) Pointrange plot showing the lateral root density in wild-type Col-0 plants, the lateral root mutant *gnom184*, the PAMP receptor mutant *fls2*, and the ethylene mutants *ein3 eil1* in response to a bacterial synthetic community (SynCom) or a natural community isolated from a natural soil. Colors represent different treatments. Significance was determined via linear modeling, letters represent the compact letter display of a Tukey post hoc test. We used two independent biological replicates per condition with 10 plants each. This experiment was repeated twice. (*F*) Pointrange plot showing the lateral root density in wild type Col-0 plants grown in full nutrient medium supplemented with 100 mM NaCl, or without Fe and colonized or not (NB) with a bacterial synthetic community or a natural community isolated from a natural soil. Colors represent different treatments. Significance was determined via linear modeling, letters represent the compact letter display of a Tukey post hoc test. We used two independent biological replicates per condition with 10 plants each. This experiment was repeated twice. (*G*) Proposed model of the mechanism used by the microbiota to regulate lateral root development.

To validate this hypothesis, we exposed wild-type and mutant plants related to ethylene signaling to seven bacterial isolates that spanned all different transcriptional profiles detected in the RNAseq experiments with or without an effect on lateral root density (Fig. 4*A*). We observed that the ethylene-insensitive mutants *ein3 eil1, ein2, and etr1* (34) significantly reduced the lateral root density in response to the selected bacteria as compared to wild-type plants (Fig. 5 *A* and *B* and *SI Appendix*, Fig. S9*A*), despite having a similar level of bacterial colonization (*SI Appendix*, Fig. S9*B*). These results indicate that an intact ethylene signaling pathway is necessary for microbiota-induced lateral root development.

Under axenic conditions, changes in ethylene signaling can disturb auxin homeostasis via inhibition of auxin biosynthesis (35). To evaluate the global effect of ethylene signaling controlling lateral root development independently of auxin biosynthesis we treated wild-type and double-mutant *ein3 eil1* plants with the auxin biosynthesis inhibitor L-Kyn. As expected, wild-type plants under axenic conditions, reduced the lateral root density upon treatment with L-Kyn. On the contrary, we noticed that the reduction in lateral root number in the *ein3 eil1* mutant was not further increased when we treated the mutant with the auxin biosynthesis inhibitor L-Kyn (*SI Appendix*, Fig. S9*C*) confirming that both ethylene signaling and auxin biosynthesis are compromised in this mutant. When the microbiota was present, wild-type plants, with intact ethylene signaling, did not reduce the lateral root density in response to L-Kyn. Nevertheless, the double-ethylene mutant *ein3 eil1* with or without L-Kyn showed a significant reduction in lateral root density as compared with wild-type plants. Therefore, these results strongly suggest that ethylene signaling and not auxin biosynthesis is necessary to respond to the microbiota effect on lateral root emergence.

Next, using the ethylene sensor *EBS:GUS,* expressing the GUS reporter under the control of a synthetic *EIN-3*-responsive promoter (36), we demonstrated the bacterial capacity to increase the level of ethylene in the root (*SI Appendix*, Fig. S9*D*). We confirmed these results in the case of RMF27, that causes the more extreme lateral root phenotype, using direct chemical quantification (*SI Appendix*, Fig. S9*E* and Dataset S5). Furthermore, the ethylene precursor 1-aminocyclopropane-1-carboxylic acid (ACC) added externally together with the bacterial isolates increases lateral root density in the *ein3 eil1* mutant, and this effect is more evident in the wild-type and lateral root mutant *arf7 arf19* and to a lesser extent in *gnom184* (Fig. 5 *C* and *D* and *SI Appendix*, Fig. S9*F*). In contrast, wild-type, ethylene mutant *ein3 eil1*, and lateral roots mutants *arf7 arf19* and *gnom184* treated with the ethylene biosynthesis inhibitor aminoethoxyvinylglycine (AVG) (37) reduced the ability to produce more lateral roots when the microbiota is present (Fig. 5 *B* and *C* and *SI Appendix*, Fig. S9*F*). The negative effect of AVG on lateral root density was not further increased in wild-type plants inoculated with the bacterial isolates in the presence of both AVG and the auxin inhibitor L-Kyn (*SI Appendix*, Fig. S9*G*). We rule out that bacteria use ACC and AVG as carbon sources (*SI Appendix*, Fig. S9*H*) and that bacteria-derived volatile compounds cause the lateral root phenotypes (*SI Appendix*, Fig. S9*I*). Altogether, these results indicate that the direct induction of ethylene signaling in the root is linked to the production of lateral roots in response to members of the plant microbiota. This mechanism can be functionally independent of auxin signaling controlling lateral root formation upon initiation and auxin biosynthesis, aspects found to be required for the regulatory role of ethylene on lateral root development described in axenic plants (18). Thus, we considered this mechanism as a microbiota-driven regulatory branch of lateral root development. Reinforcing this, we observed the same effect in *Selaginella* treated with ACC or AVG alone or with the bacterial isolate RMF27 (*SI Appendix*, Fig. S9*J*).

We noticed a negative correlation between the induction of defense genes and lateral root development in our RNAseq analysis (Fig. 4*A* and *SI Appendix*, Fig. S7 *D*–*F*). Thus, we asked whether the suppression of the plant immune system is required for the induction of lateral root formation by the microbiota. We evaluated the capacity of a set of seven bacterial isolates, able to produce different lateral root phenotypes, to induce the expression of 11 defense marker genes (38, 39) in wild-type plant roots and shoots using RT-PCR. We did not find significant differences in the expression of these genes in roots and shoots across the bacterial strains used (*SI Appendix*, Fig. S10*A*) nor did we find a correlation between gene expression levels and lateral root density (*SI Appendix*, Fig. S10*B*). This indicates that the ability to produce lateral roots is not strictly linked to changes in the activation of the plant immune system driven by bacterial colonization.

Furthermore, high levels of ethylene biosynthesis can be achieved during the PAMP-induced immune response via the Yang cycle (40). We quantified the lateral root density in the *mtk1* and *fls2* mutants important for the ethylene synthesis in the Yang cycle and PAMP perception, respectively, in response to bacterial isolates (40). We observed no significant differences in lateral root density between wild-type plants and both mutants in response to the presence of bacteria (*SI Appendix*, Fig. S10*C*). This suggests that the high level of ethylene production upon activation of the plant immune system might not be necessary for lateral root development in response to bacterial isolates.

### Microbiota Controls Lateral Root Development under Stress.

We studied whether microbial control of lateral root formation is present in plants colonized with more complex microbiota. We inoculated wild-type plants, lateral root mutant *gnom184*, the ethylene double-mutant *ein3 eil1,* and the PAMP receptor *fls2,* with both a 16-member bacterial synthetic community whose individual members can change root branching, and a microbiome extracted from a natural soil (6). Wild-type plants, *gnom184*, and *fls2* produced more lateral roots when inoculated with the synthetic and natural microbiota as compared to noninoculated plants (Fig. 5*E*). In contrast, the ethylene mutant *ein3 eil1* showed no significant differences in lateral root density in response to complex microbiota (Fig. 5*E*). Therefore, we recapitulated our previous observations indicating that the plant microbiota can control aspects of lateral root development via ethylene response induction in the root.

Finally, we tested whether the plant microbiota is able to regulate root branching under different abiotic stresses. We inoculated wild-type Col-0 plants with both synthetic and natural communities under high NaCl and low Fe, stresses that do not favor the development of lateral roots (41, 42). In all cases, inoculated plants produced more lateral roots as compared to the uninoculated plants (Fig. 5*F*). However, in the case of salt stress, the magnitude of lateral root changes found in response to both microbial communities relative to axenic conditions was significantly lower compared to plants grown with sufficient nutrients (*SI Appendix*, Fig. S10*D*), indicating that the lateral root developmental program can integrate not only the microbial effect but also environmental fluctuations. Therefore, the microbiota-enhancing effect on lateral root development increases root plasticity, which is necessary for plants to better adapt to natural ecosystems where abiotic stresses are frequent.

## Discussion

In terrestrial plants, the emergence of roots represents a decisive evolutionary event that facilitated the colonization of the terrestrial environment. This process has been assisted by the plant microbiota through a variety of coevolved mechanisms. For example, the effect of the bacterial genus *Variovorax* on the development of primary roots (7) and of the arbuscular mycorrhizal fungi on lateral root formation (8) have been described. Here, we have established that members of the plant microbiota largely control root architecture in vascular plants, inducing a myriad of modifications in root morphology, especially root branching, an effect that is conserved in basal land plants.

The effect of root microbiota on mineral nutrient accumulation has been previously associated with the microbial capacity to change the deposition of root diffusion barriers in the endodermis, a specialized root cell layer controlling nutrient and water uptake (6). We clarified here that the microbial effect on lateral root development is independent of the deposition of the root diffusion barrier suberin. Further, we provided evidence that the microbiota control on root branching is not directly link to mechanisms regulating lateral root development in response to nutrient availability (14).

We discovered that the microbiota can exert control over some stages of root branching through the induction of the phytohormone ethylene response in the root. This ethylene response induction, in the case of the commensal bacteria analyzed in this work, does not require activation of the plant immune system. These observations separate the mechanism of lateral root formation described here from other developmental responses in the plant associated with the activation of the plant immune system upon microbial detection (8, 39).

Furthermore, we provided evidence that this microbiota-driven mechanism influences the early stages of lateral root development, after the formation of the prebranch sites, increasing the number of primordia that develop into new lateral roots. Importantly, this positive microbial effect on lateral root formation can be functionally independent of the auxin network that controls lateral root developmental events upon initiation and auxin biosynthesis (Fig. 5*G*). These characteristics differentiate this microbiota-driven mechanism from the known ethylene-auxin crosstalk involved in lateral root development under axenic conditions (18). Therefore, we validated in plants colonized by microbes, previous indications found in axenic conditions, that lateral root development might involve other components that are independent of auxin signaling (15).

All these features, absent in mechanisms of lateral root development previously described (8, 14), position our microbiota-driven pathway as an independent branch in the endogenous regulatory system of lateral root development in plants. We theorize that although the activation of ethylene is sufficient to control the microbiota effect on lateral roots, auxin, nutrients, and the immune system may be influencing indirectly the microbiota-driven pathway, and these interactions among distinct regulatory branches contribute to the integration of environmental cues, both biotic and abiotic, into the endogenous developmental program to modulate lateral root plasticity.

Therefore, our discoveries expose a new alternative mechanism of root branching regulation, driven by the plant microbiota, which is of great relevance for root branching plasticity in natural ecosystems when microbes are omnipresent. Our findings significantly advance our knowledge on how plants integrate microbial function into mechanisms of root branching into a broad evolutionary context. The design of microbial-based strategies is of critical importance for the optimization of the shape of the root system to increase its capacity for water and mineral nutrients uptake, plant anchorage, and an optimal interaction with soil microbiota. Furthermore, our discovery could guide future microbial-based solutions to increase food production in eroded, nutrient-poor, and compacted soils, where plant performance relies on root function.

## Materials and Methods

Detailed descriptions of all utilized methods and data analysis workflows can be found in *SI Appendix*.

### Screen of Bacterial Isolates Based on Their Ability to Change Root Architecture.

For all bacterial isolates analyzed, bacterial strains were cultured axenically, washed using 10 mM MgCl_2_, and prepared at a final concentration of 10^5^ c.f.u/mL. The individual cultures (100 μL) were spread on the surface of square agar plates before transferring the seedlings. Then, 10 7-d-old seedlings were transferred to the agar plate inoculated with the individual isolates. After 7 d, we determined root architecture-related features.

### Determination of Root Architecture Parameters.

We quantified 21 features related to root architecture using the SmartRoot software (43).

### Identification of Auxin-Related Operons.

We used the literature-curated biosynthetic and degrading pathways described in Metacyc (44) to identify Clusters of Orthologous Genes and Kyoto Encyclopedia of Genes and Genomes orthology identifiers of key enzymes involved in the biosynthesis and degradation of auxin.

### Individual Bacterial Effects on *Selaginella* and Lateral Root Mutants.

Fresh-cut *Selaginella* explants of similar sizes lacking roots were transferred to each of the individual-strain inoculated plates (100 μL at 10^5^ c.f.u/mL). After 14 d, the number of bifurcation events was counted.

Seven-day-old *Arabidopsis* lateral root mutant (*iaa14 slr-1*, *arf7 arf19*, *nph4-1*, *lbd16-1, gnom184*, *gelpquint1*, and *gelp72*) seedlings were transferred to each of the individual-strain inoculated plates (100 μL at 10^5^ c.f.u/mL). After 7 d, the density of lateral roots was determined.

### Effect of Nutrient Deficiency on Lateral Root Development.

Seven-day-old seedlings of lateral root mutants (*arf7 arf19*, *nph4-1*, *lbd16-1, gnom184*) and Col-0 were transferred to agar plates containing 0.5X MS, 0.005X MS, and 0.0005X MS inoculated with 100 μL of 10 mM MgCl_2_. After 7 d, the concentration of mineral nutrients in plant shoots was determined using Inductively Coupled Plasma Mass Spectrometry (ICP-MS).

### Microbiota Effect on Lateral Root Formation.

The microbiota effect on lateral formation was studied in the lateral root primordia marker lines *pSKP2B::GUS* line (23) and *pLBD16:GFP* (24), the pre-branch site marker line *DR5:Luciferase* (15), and the auxin sensor *DII:VENUS* (25) grown on agar plates for 6 d.

To determine the effect of auxin signaling and transport on lateral root development in the presence of the microbiota, we transferred 7-d-old Col-0 and lateral root mutant seedlings to agar plates supplemented with 5 μM NPA (Sigma) or 1.5 μM L-Kyn (Sigma) and inoculated or not with the bacterial isolates. After 7 d, the lateral root density was determined.

### Mineral Nutrient Analysis.

The concentration of mineral nutrient analysis in plant shoots was determined using ICP-MS according to (6).

### In Vitro Auxin Synthesis and Degradation Assays.

For IAA synthesis, we used a modified method from (45). In each culture, IAA was determined using a modified version of the Salkowski method (46) by measuring the absorbance at 530 nm using a FLUOstar® Omega (BMG LABTECH).

For the IAA degradation assays, bacterial isolates were grown in M9 minimal salts medium (Sigma) supplemented with 2 mM MgS0_4_, 0.1 mM CaCl_2_, 10 μM FeSO_4_, and either 0.4 mM IAA or 15 mM succinate (Sigma) as carbon sources. IAA was determined as above.

### Lateral Root Primordia Quantification.

To visualize and quantify the number of lateral root primordia, 6-d-old seedlings roots were fixed, cleared, and quantified using a Leica 2 DM5000B fluorescence microscope at 40× magnification.

### Prebranch Sites Quantification.

For the visualization of *DR5:Luciferase* activity, plates containing 6-d-old *DR5: Luciferase* seedlings exposed or not to the different bacterial treatments were sprayed with 5 mM Beetle Luciferin (Promega) and then imaged for 4 min using a Lumazone CA Automated Chemiluminescence System (Roper Bioscience).

### Quantification of Changes in Auxin Signaling.

To determine whether the bacteria isolates change the auxin signaling in the root, we used the auxin sensor *DII:VENUS* (25). VENUS expression was visualized using a Leica SP8 confocal microscope, 40× objective.

### NPA and L-Kyn as Bacterial Carbon Sources.

We grew the bacterial isolates in M9 minimal salts medium (Sigma) supplemented with 2 mM MgS0_4_, 0.1 mM CaCl_2_, 10 μM FeSO_4_, and either 50 μM NPA or 15 μM L-Kyn, or 15 mM succinate (Sigma) as carbon sources for 2 d at 28 °C with agitation (250 rpm). In all cases, the bacterial growth was monitored by measuring the optical density (OD) at 600 nm using a FLUOstar® Omega (BMG LABTECH).

### Experiments to Define the Molecular Mechanism Coordinating Plant Microbiota and Root Branching.

Seven-day-old Col-0, lateral root mutants (*arf7 arf19*, *nph4-1*, *lbd16-1*, and *gnom184*), ethylene mutants (*ein3 eil1, ein2, etr1, ctr1*, *eto3, and mtk1*), and defense mutant *fls2* seedlings were transferred to 0.5X MS agar plates inoculated with the individual isolates alone or plates supplemented with 5 μM NPA or 1.5 μM L-Kyn, or 10 μM of the ethylene precursor 1-aminocyclopropane-1-carboxylate (ACC), or 2 μM of the ethylene biosynthesis inhibitor AVG (37) or plates inoculated with bacterial strains together with one or two of the chemicals described. After 7 d, we quantified the lateral root density.

### ACC Quantification.

ACC was extracted and quantified according to Bulens et al. (47) using gas chromatography (Shimadzu GC2014).

### Ethylene Response in *Selaginella*.

Fresh-cut *Selaginella* explants of similar sizes, lacking roots, were transferred to agar plates with 0.5× MS inoculated with RMF27 or to plates supplemented with 10 μM ACC, or 2 μM AVG or combinations of them. After 14 d, the number of bifurcation events was quantified.

### RNA Extraction.

In all cases, RNA was extracted from plant roots and shoots following Logemann et al. (48).

### Real-Time PCR Analysis.

For RT-PCR analysis, all RNA samples were first DNase treated with DNAse I (Thermo Scientific). cDNA synthesis was performed using the RevertAid First Strand cDNA Synthesis Kit (Thermo Scientific) according to the manufacturer’s recommendations. Gene expression was determined using the SensiMix™ SYBR® Hi-ROX Kit (Bioline).

### Plant RNA Sequencing.

RNA libraries were prepared according to (6). Each library pool was sequenced on three lanes on an MGI Tech MGISEQ-2000 sequencing platform at Beijing Genomics Institute, Shenzhen, China.

### Bacterial Colonization Analysis.

To reisolate and quantify bacteria strains across the different treatments, we used the colony-forming unit (c.f.u) method according to (6).

### ACC and AVG as Bacterial Carbon Sources.

We grew the bacterial isolates in M9 minimal salts medium (Sigma) supplemented with 2 mM MgS0_4_, 0.1 mM CaCl_2_, 10 μM FeSO_4_, and either 100 μM ACC or 20 μM AVG, or 15 mM succinate (Sigma) as carbon sources. All cultures were grown for 2 d at 28 °C with agitation (250 rpm). In all cases, the bacterial growth was monitored by measuring the OD at 600 nm using a FLUOstar® Omega (BMG LABTECH).

### Split Root Experiments.

To determine the effect of the volatile compounds produced by the bacteria isolates on lateral root formation, we performed split root assays. For each bacterium, we designed four treatments: (plant + no bacteria) vs. (no plant + no bacteria); (plant + bacteria) vs. (no plant + bacteria); (plant + bacteria) vs. (no plant + no bacteria); and (plant + no bacteria) vs. (no plant + bacteria). We determined the primary root length and the number of secondary roots across all conditions from images taken using a linear robot camera.

### Natural Microbiota Isolation from Soil.

Soil natural microbial populations were isolated from a natural soil from Sutton-Bonington Campus (University of Nottingham, UK; +52° 49′ 59.75″N, −1° 14′ 56.62″W) according to (6).

### Bacterial Synthetic Community Preparation.

The bacterial synthetic community was designed using 16 bacterial strains that cover the diversity of the bacterial effect on lateral root development. Bacterial isolates were grown, cleaned, and mixed according to (6).

### Abiotic Stresses.

To impose the stresses to the plants, the composition of the 0.5× MS medium was amended according to (6).

## Supplementary Material

Appendix 01 (PDF)

Dataset S01 (XLSX)

Dataset S02 (XLSX)

Dataset S03 (XLSX)

Dataset S04 (XLSX)

Dataset S05 (XLSX)

Dataset S06 (XLSX)

Dataset S07 (XLSX)

## Data Availability

RNA-Seq raw sequence data are available at the NCBI Gene Expression Omnibus under accession no. GSE210742. All data and code needed to reproduce all analyses can be found at https://github.com/isaisg/rootbranchingmicro. Previously published data were used for this work (Part of the data used to generated the *SI Appendix*, Fig S4*E* was previously published in ref. 6).
