# Supplementary material for: Plant microbiota controls an alternative root branching regulatory mechanism in plants
Source: Proc Natl Acad Sci U S A. 2023 Apr 3;120(15):e2301054120. doi: 10.1073/pnas.2301054120 (PMC10104509; doi:10.1073/pnas.2301054120)
Supplement: Supplementary file 1 — Appendix 01 (PDF) [file pnas.2301054120.sapp.pdf]

Supporting Information for

Plant microbiota controls an alternative root branching regulatory mechanism in plants

Mathieu Gonin[a,1], Isai Salas-González[b,1], David Gopaulchan[a], Juan P. Frene[a,c], Stijn Roden[d], Bram Van de Poel[d,e], David E. Salt[a,c], and Gabriel Castrillo[a,c,2].

[a]School of Biosciences, University of Nottingham, LE12 5RD, United Kingdom.
[b]Center for Genomics Sciences, Universidad Nacional Autónoma de México, 04510 Mexico City, Mexico.
[c]Future Food Beacon of Excellence, University of Nottingham, LE12 5RD, United Kingdom.
[d]Division of Crop Biotechnics, Department of Biosystems, KU Leuven, 3001 Leuven, Belgium.
[e]Leuven Plant Institute, KU Leuven, 3001 Leuven, Belgium.

[1]M.G. and I.S.-G. contributed equally to this work.
[2]To whom correspondence may be addressed. Email: gabriel.castrillo@nottingham.ac.uk.

This PDF file includes:

Figures S1 to S10
Dataset S1 to S7
Detailed Material and Methods
Data availability
SI References

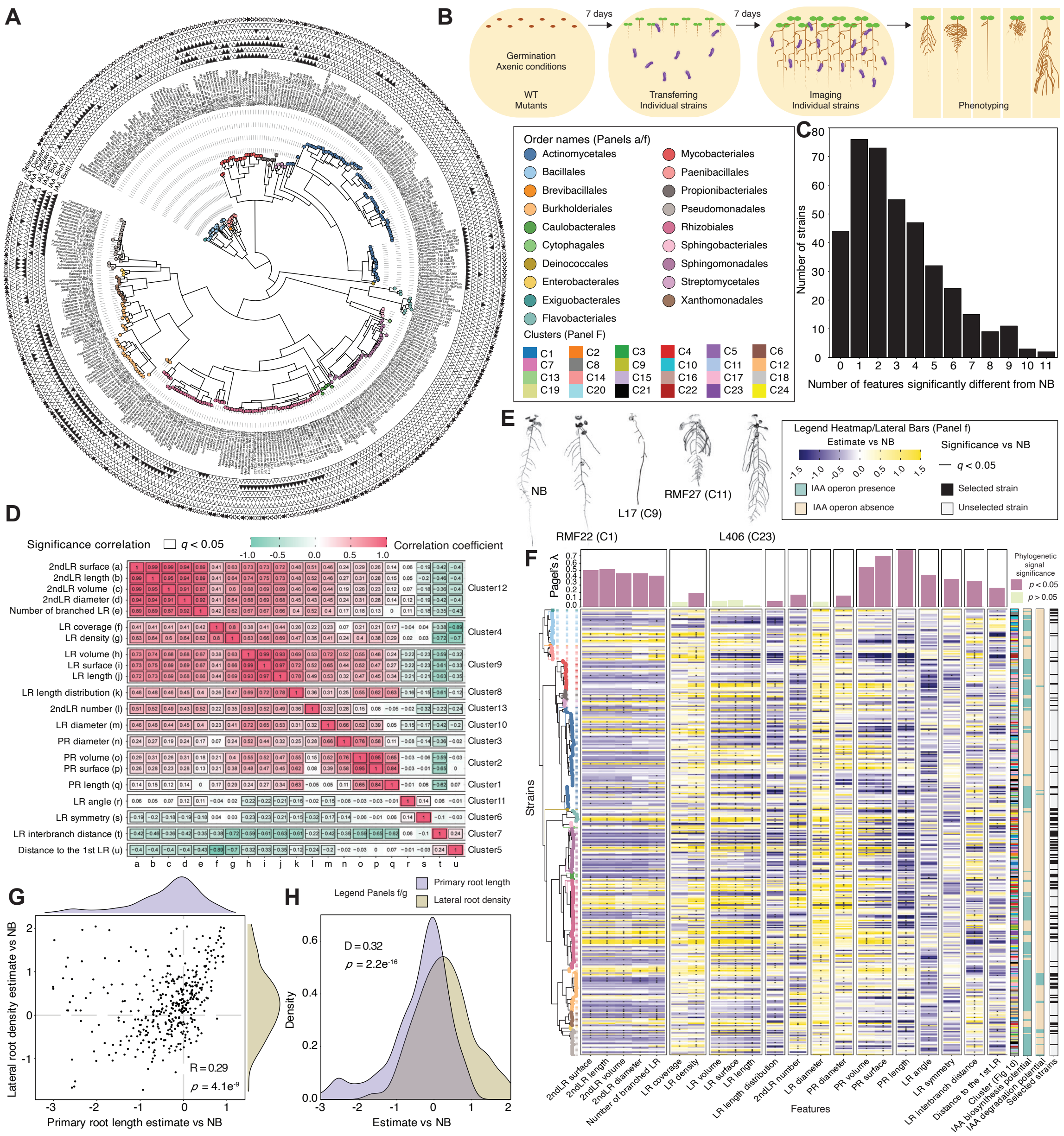

***SI Appendix,* Fig. S1. Individual bacterial isolates change root architecture. (*A*).** Phylogenetic tree built using the genome of 373 bacterial strains available for the 391 isolates belonging to the bacterial collection used in this work. Tree has the tips colored according to the bacterial taxonomic class. From the inside to the outside, different rings represent the presence of known auxin biosynthetic operons (IAA_BioIII, IAA_BioIV, IAA_BioV, IAA_BioVI), auxin degrading operons (IAA_Deg*lac*, IAA_Deg*lad*) in the bacterial genomes, and the bacterial isolates that were selected (selected) for further experiments. **(*B*).** Scheme of the experimental pipeline used to determine the effect of individual bacterial strains on the root architecture features quantified in this work. See material and methods section for details about the protocol used. **(*C*).** Bar graph showing the number of bacterial isolates, out of the 391 analyzed, able to significantly modify one or multiple root architecture features. **(*D*)**. Heatmap showing the Pearson's correlation coefficient between the different root architecture features quantified in response to the individual bacterial isolates collection. For each pairwise comparison between features, the correlation coefficient is shown. Features have been hierarchically clustered according

to the correlation coefficient. Significant correlations (*q*-value < 0.05) are outlined in black. Notice that some features are highly correlated indicating certain level of redundancy among the root parameters analyzed. See materials and methods section for the root architecture features definition**. (*E*).** Exemplary images of changes in plant root architecture induced by individual members of the plant microbiota. The plants have been exposed to different bacteria, whose names appear at the *Top* of the figure, for seven days or grown under axenic conditions (NB). The number of the cluster, defined in Fig. 1*D*, to which each bacterium belongs is in parentheses. **(*F*).** Heatmap showing the bacterial strains' estimated effect on individual root architecture features with respect to the no bacteria control. Significantly different values from the no bacteria control are highlighted with a small horizontal line. Rows (Strain) of the heatmap are sorted according to the bacterial collection's phylogeny, as indicated by the tree on the *Left*. Columns (root features) are sorted based on the hierarchical clustering analysis using all vs all pairwise correlation comparison presented on panel ***D***. Bar graph on the *Top* of the panel shows the λ-values from Pagel's λ test for phylogenetic signal. Significant ($p < 0.05$) or not ($p > 0.05$) λ-values are colored in pink and grey, respectively. Colors on the vertical bars on the *Right* of the panel, from *Left* to the *Right*, represent the clusters from Fig. 1*D*, the presence of known auxin (IAA) biosynthetic operons, IAA degrading operons, and the bacterial strains selected (selected) for further experiments, respectively. **(*G*).** Scatterplot showing the correlation between the estimated effects induced by the bacterial collection over the lateral root density and the primary root. Each dot in the scatterplot represents the effect of a bacterial strain. The inter-feature Pearson's correlation coefficient R and its associated *p*-value are shown in the figure. At the *Top* of each axis are shown the corresponding data distribution. **(*H*).** Distribution of the bacterial magnitude effects on primary root length and lateral root density. The graph shows the calculated effect sizes distribution for all bacterial isolates versus no-bacteria control across the two features analyzed. The result of a Kolmogorov-Smirnov test (D) and its associated *p*-value used to determine the difference between distributions are shown. For this experiment we used at least two independent biological replicates per bacterial condition with 10 plants each.

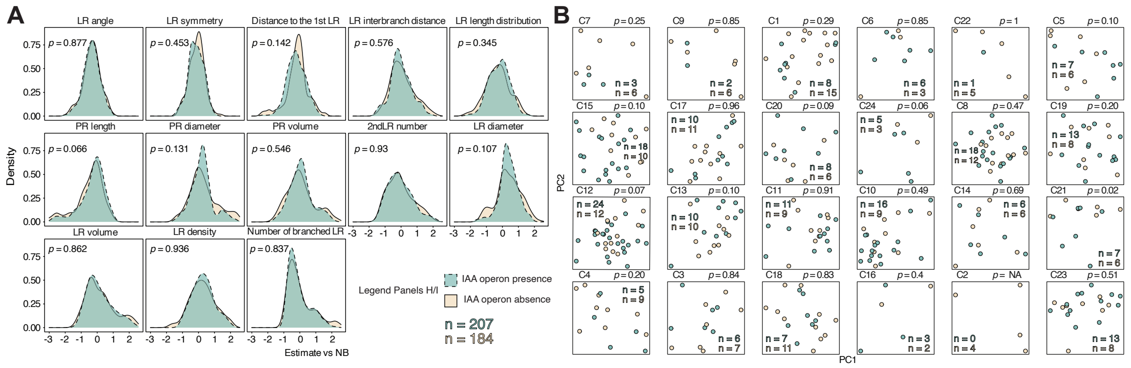

***SI Appendix,* Fig. S2. Members of the plant microbiota might modify root development independently of their ability to change root auxin homeostasis. (*A*).** Distribution of the bacterial magnitude effects on individual root architecture features. The graphs show the calculated effect size (estimates) distribution for the set of bacteria bearing known auxin-related operons (IAA operon presence) or not (IAA operon absence) versus no-bacteria control across individual root features. The difference (*p*-value) between the distributions was calculated with a Kolmogorov-Smirnov test. The total number of strains with or without IAA operons are shown **(*B*).** Scatterplots showing, for each cluster of strains (Fig. 1*D*), the projection of a Principal component analysis (PCA) utilizing the root architecture features within cluster. Dots in the projection represent individual strains that are colored based on the presence of auxin-related operons within its genome. For each cluster, differences between the two groups of strains (IAA operon presence and IAA operon absence) were estimated via PERMANOVA. The PERMANOVA *p*-value and the number of the strain cluster (C) from Fig. 1*D* are shown on the *Top* of each panel. In all cases, the total number of strains analyzed with or without IAA operons is shown.

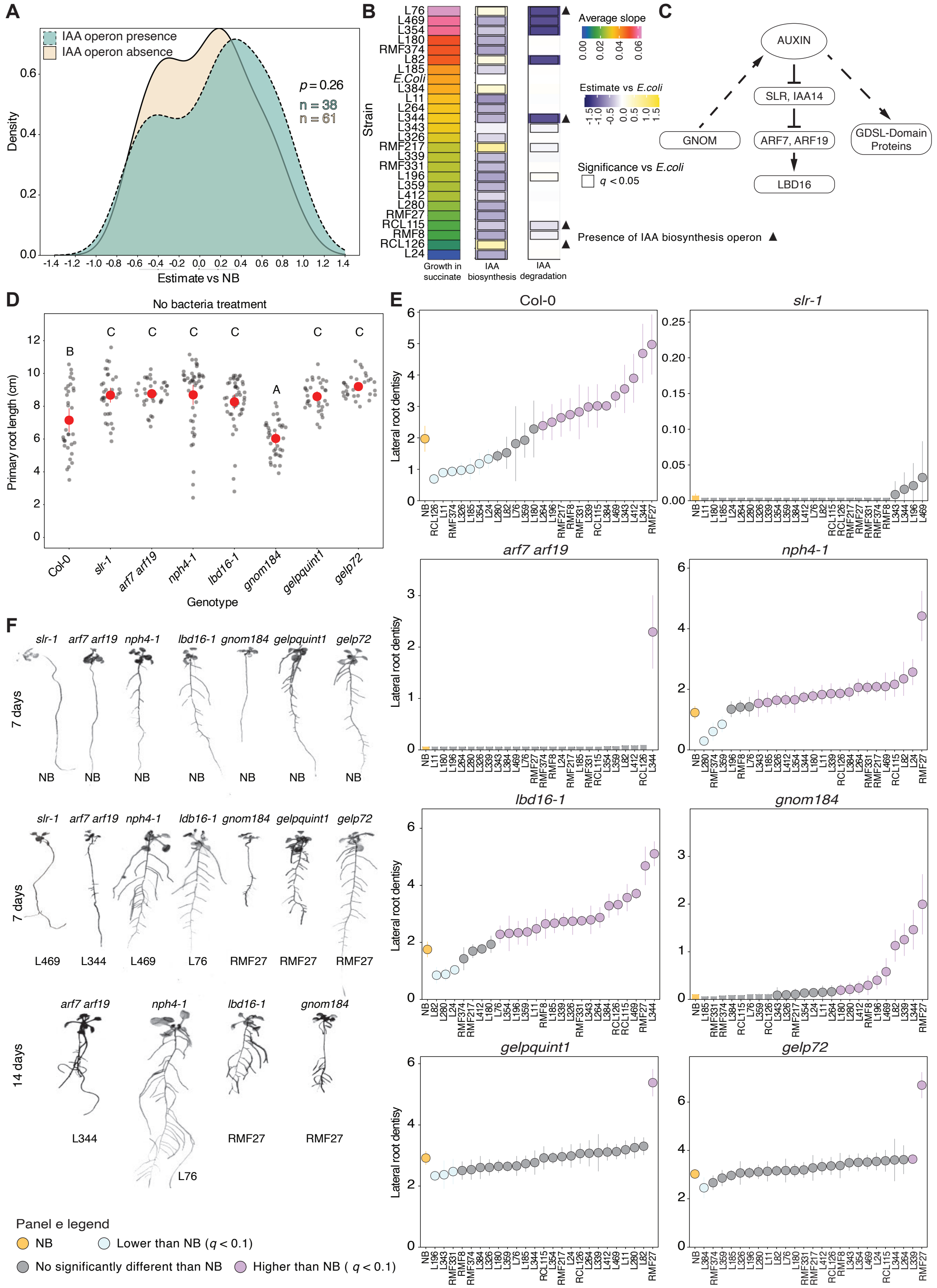

***SI Appendix,* Fig. S3. The microbiota effect on some stages of lateral root development can be auxin independent. (*A*).** Distribution of the bacterial magnitude effect on *Selaginella moellendorffii* (*Selaginella*) root bifurcation. The graphs show the calculated effect sizes (estimates) distribution for the set of bacteria bearing known auxin-

related operons (IAA operon presence) or not (IAA operon absence) versus no-bacteria control in *Selaginella* root bifurcation. The difference (*p*-value) between the distributions was calculated with a Kolmogorov-Smirnov test. The number of strains with or without known IAA operons are shown. **(*B*).** In vitro synthesis and degradation of IAA by the bacterial strains used. From *Left* to the *Right*, the heatmap shows the slope of the exponential phase of the growth curves of different bacterial isolates grown in M9 minimal salts medium supplemented with succinate (growth in succinate), the estimate values of IAA concentrations relative to those found for *Escherichia coli* used as a control (IAA biosynthesis), and the estimate values of IAA concentrations relative to those found for *Escherichia coli* (IAA degradation). In the figure, the triangle denotes presence of IAA biosynthesis operon in the bacterial genome. For this experiment we used three independent biological replicates and the experiment was repeated three times. **(*C*).** Reduced diagram of lateral root development regulation in *Arabidopsis thaliana.* Dashed lines indicate undetermined direct relationships. Auxin controlled regulatory modules are successively activated during lateral root formation. SLR/IAA14–ARF7–ARF19 module controls the starting process in lateral root founder cells. The LBD16, a transcription factor, regulates cell cycle genes important for the formation of the lateral root primordia (*1*). GNOM controls the balance between esterified and de-esterified pectin, a cell wall component, required for a proper initiation of lateral root primordia (*2*). Auxin-induced GDSL-motif containing enzymes (for example GELP72) regulate suberin deposition involved in lateral root emergence (*3*). **(*D*).** The graph shows the primary root length of wild-type Col-0 plants and the lateral root mutants *iaa14 slr-1*, *arf7 arf19*, *nph4-1*, *lbd16-1, gnom184*, *gelpquint1,* and *gelp72* after seven days of colonization with different bacterial isolates. Notice that, in general, under full nutrient conditions, the length of the primary root in the mutant genotypes used is greater than in wild-type plants. **(*E*).** Analysis of lateral root density in wild-type plants Col-0 and lateral root mutants *iaa14 slr-1*, *arf7 arf19*, *nph4-1*, *lbd16-1, gnom184*, *gelpquint1*, and *gelp72*, after seven days of colonization with different bacterial isolates that change lateral root development. The name of the plant genotype is on the top of each graph. In yellow is the no bacterial (NB) control in each case. This data was used to generate Fig. 2*C*. For this experiment we used two independent replicates per bacteria and genotype condition with 10 plants each. **(*F*).** Exemplary images of wild-type Col-0 plants and lateral root mutants *iaa14 slr-1, arf7 arf19*, *nph4-1*, *lbd16-1, gnom184*, *gelpquint1*, and *gelp72* grown in axenic MS plates (No bacteria (NB)) or MS plates inoculated with bacterial isolates that induce changes in lateral root density. To make the bacterial effect on lateral root formation clear, some mutants were imaged at 7 d and others at 14 d after inoculation with the bacteria.

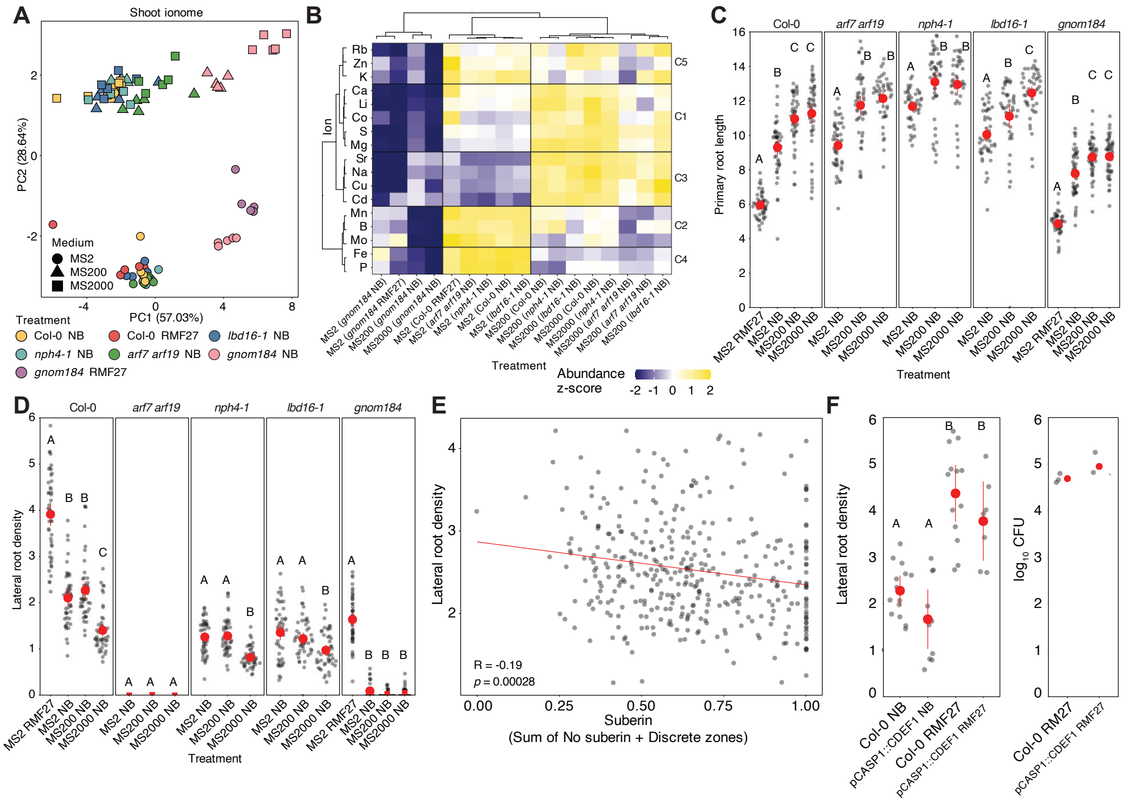

SI Appendix,* Fig. S4. The root microbiota controls lateral root development independently of the nutritional homeostatic mechanisms of the plant. (*A*).** Principal component analysis (PCA) of mineral nutrient composition showing the mineral nutrient concentration projection of wild-type plants and lateral root mutants *arf7 arf19, nph4-1, ldb16-1, gnom184* grown on axenic agar plates containing 0.5x MS (MS2), 0.005x MS (MS200), and 0.0005x MS(MS2000) solidified with 1% agar. As a control we analyzed the mineral nutrient concentration of wild-type plants and the mutant *gnom184* inoculated with the bacterium RMF27 that cause the highest lateral root density. In the scatterplot, each color represents a treatment and the geometrical shapes the MS medium dilution. **(*B*).** Heatmap showing the mineral nutrient concentrations in shoots of wild-type plants and lateral root mutants *arf7 arf19, nph4-1, ldb16-1, gnom184* grown on axenic agar plates containing 0.5x MS (MS2), 0.005x MS (MS200), and 0.0005x MS (MS2000) solidified with 1% agar. As a control we analyzed the mineral nutrient concentrations in wild-type plants and in the mutant *gnom184* inoculated with the bacterium RMF27. The values were clustered according to the treatments and mineral nutrient concentration. Note that the level of mineral nutrient concentrations in wild-type and the mutants grown under axenic low nutrient conditions are similar or lower than the wild-type and mutant plants inoculated with RMF27. **(*C*).** Pointrange plots showing the primary root length and **(*D*).** lateral root density in wild-type plants and lateral roots mutants *arf7 arf19, nph4-1, ldb16-1, gnom184* grown on axenic agar plates containing 0.5x MS (MS2), 0.005x MS (MS200), and 0.0005x MS(MS2000) solidified with 1% agar. As a control we analyzed the lateral root density of wild-type plants and the mutant *gnom184* inoculated with RMF27. Note that low nutrient concentration (Panel ***A and ***B***) did not activate lateral root formation in wild-type and mutants grown under axenic conditions to levels found in plants inoculated with bacteria. For this experiment we used five independent biological replicates. **(*E*).** Pearson correlation analysis between lateral root density from Fig. 1 and the distance from the root

tip to the continuous zone of suberization in the root extracted from (*4*) in plants exposed to a collection of 391 individual bacterial isolates. The correlation coefficient R and its *p*-value are shown. **(*F*).** (*Left*) Pointrange plots showing the lateral root density in wild-type plants and the line *pCASP1::CDEF1* that expresses the cuticle destructing factor1 that degrades suberin (*5*) grown in axenic conditions (NB) or exposed to the bacterium RMF27 that caused the highest lateral root density phenotype. (*Right*) The colonization capacity of RMF27 was determined in roots of wild-type plants and the *pCASP1::CDEF1* line using log-transformed-colony forming units. The red dots represent the average value of the samples. Note that less endodermal suberization does not affect the high lateral density induced by the bacterium RMF27. We used two independent biological replicates with 10 plants each.

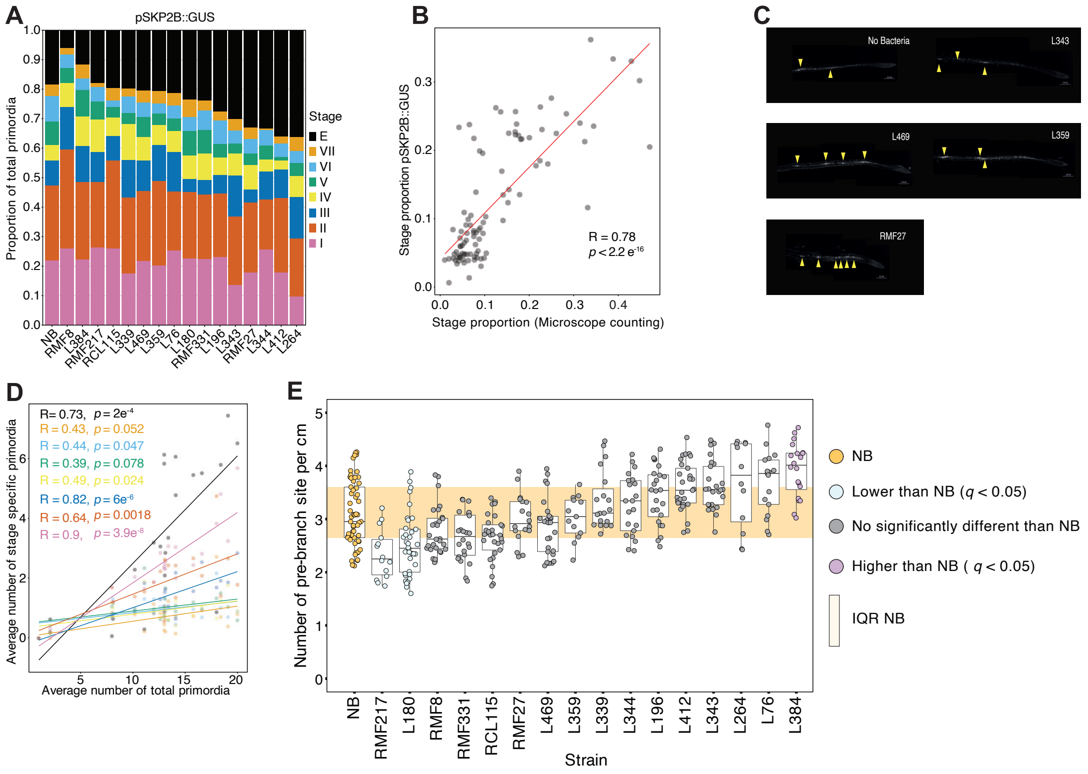

***SI Appendix,* Fig. S5. Following the formation of pre-branch sites, the root microbiota increases the number of early-stage primordia capable of developing into a lateral root. (*A*).** Stacked bar charts showing the proportion of lateral root primordia in the different developmental stages counted using the *pSKP2B::GUS* line (*6*), a reporter line for lateral root primordia formation, inoculated with a collection of bacterial isolates. Different colors in the chart represent the different lateral root primordia stages (I, II, III, IV, V, VI, VII, and emerged (E)) (*1*). For this experiment we used 10 plants per bacterial condition. **(*B*).** Scatterplot showing the Pearson correlation analysis between the number of primordia counted directly from the wild-type plants (primordia counting microscope) and the primordia number determined using the *pSKP2B::GUS* line (*6*). The correlation coefficient R and its *p*-value are shown. **(*C*).** Exemplary images of lateral root primordia detected using the reporter line *pLBD16:GFP* (*7*), a marker for lateral root primordia, grown in axenic MS plates (No bacteria) or MS plates inoculated with different bacterial isolates. Arrows point to the lateral root primordium in the root. **(*D*).** Scatterplot showing the Pearson correlation analysis between the total number of primordia per root and the number of primordia at different stages from Fig. 3*A*. The correlation coefficient R and its *p*-value are shown in each case**.** Different colors denote different primordia developmental stages. **(*E*).** Boxplots showing the density of pre-branch sites in roots of *DR5:Luciferase* seedlings exposed to 16 different bacterial isolates (Strain). In the graph, no bacteria (NB) treatment is colored in orange and the horizontal shading is the interquartile range (IQR) of pre-branch density in plants grown under axenic conditions. Values colored in purple and blue are significantly higher or lower from the no bacteria (NB) control (Kruskal-Wallis test, $q < 0.05$), respectively. Values colored in grey are no significantly different from the no bacteria (NB) control. We used two independent biological replicates per bacterial condition with 10 plants each.

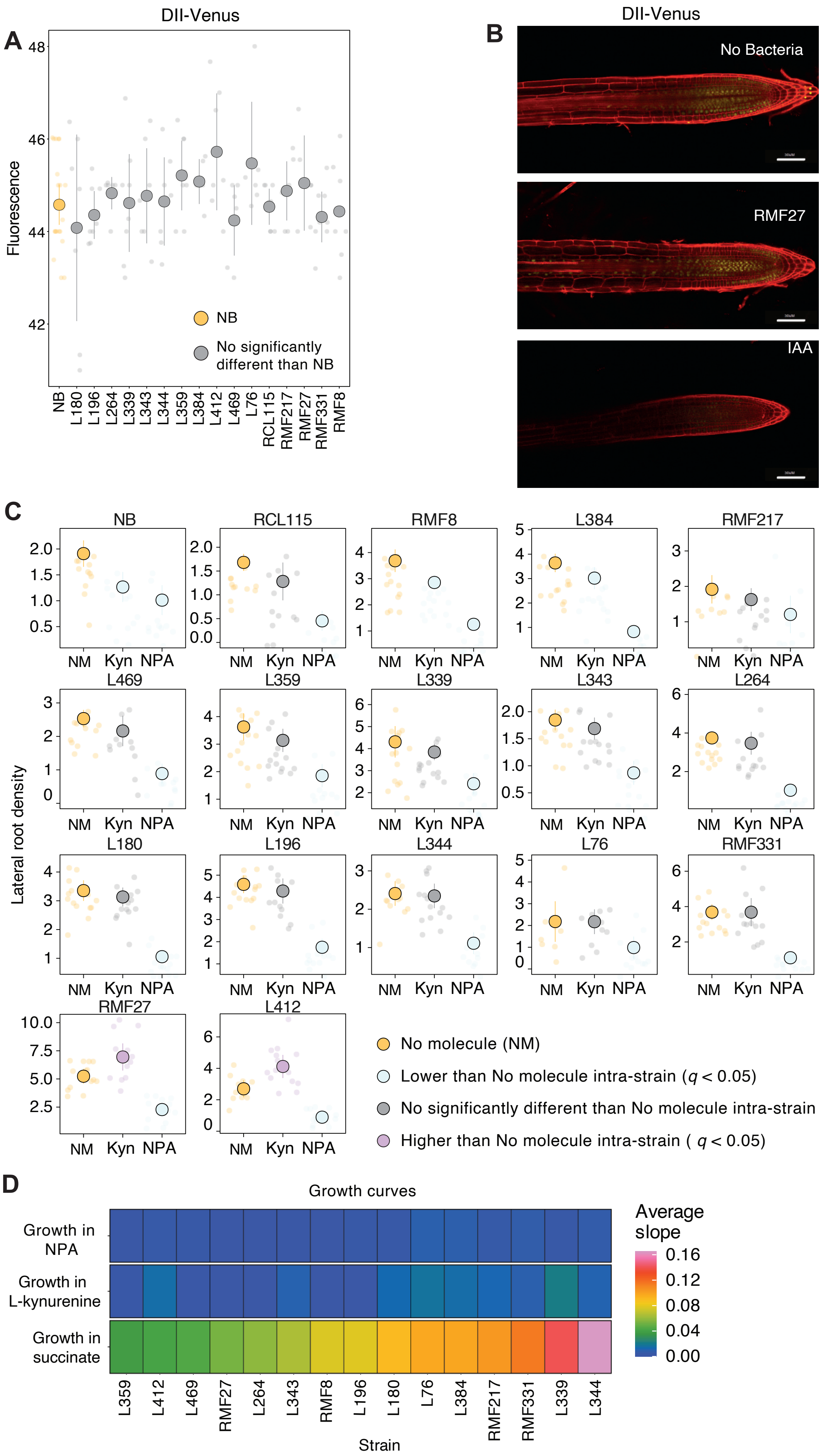
A
DII-Venus
Fluorescence
48
46
44
42
NB
No significantly different than NB
NB L180 L196 L264 L339 L343 L344 L359 L384 L412 L469 L76 RCL115 RMF217 RMF27 RMF331 RMF8
B
DII-Venus
No Bacteria
RMF27
IAA
C
Lateral root density
NB
RCL115
RMF8
L384
RMF217
L469
L359
L339
L343
L264
L180
L196
L344
L76
RMF331
RMF27
L412
NM Kyn NPA
No molecule (NM)
Lower than No molecule intra-strain (q < 0.05)
No significantly different than No molecule intra-strain
Higher than No molecule intra-strain (q < 0.05)
D
Growth curves
Growth in NPA
Growth in L-kynurenine
Growth in succinate
Average slope
0.16
0.12
0.08
0.04
0.00
L359 L412 L469 RMF27 L264 L343 RMF8 L196 L180 L76 L384 RMF217 RMF331 L339 L344
Strain

***SI Appendix,* Fig. S6. Once the pre-branch sites have been formed, the root microbiota can control lateral root primordia progression via a mechanism that can be independent of auxin biosynthesis. (*A*).** The graph shows the fluorescence quantification in the root tip of *DII:VENUS* plants, an auxin sensor, inoculated with different bacterial isolates capable of inducing changes in the density of lateral roots. In the *DII:VENUS* line, the intensity of the fluorescence is inversely proportional to the concentration of auxin in the root. Note that the bacterial isolates used did not significantly alter the auxin levels in the root. For this experiment we used two independent replicates per bacteria condition with at least 5 plants each. **(*B*).** Exemplary pictures of the fluorescence found in the *DII:VENUS* sensor grown in axenic MS plates (No bacteria), MS plates supplemented with 100 nM IAA (Indole-3-acetic acid), or in the presence of the bacterium RMF27 that induced the higher lateral root density. **(*C*).** Analysis of lateral root density in wild-type plants inoculated with different bacterial isolates alone (no molecule) or together with L-kynurenine (Kyn) or N-1-naphthylphthalamic acid (NPA), an auxin biosynthesis and auxin polar transport inhibitors, respectively. As a control we used wild-type plants grown axenically (NB) and treated or not (no molecule, NM) with L-kynurenine (Kyn) or N-1-naphthylphthalamic acid (NPA). We evaluated the molecule treatment effect for each strain independently (Colors within boxes). The name of the bacterial treatment used is on *Top* of each graph. See also Fig. 3*B*. Notice that lateral root density in plant colonized by bacteria is robust to L-kynurenine treatment but not to NPA, indicating that the root microbiota controls lateral root development through a mechanism that can be independent of auxin biosynthesis. For this experiment we used two independent biological replicates with 10 plants each. **(*D*).** The heatmap shows the slope of the exponential phase of the growth curves of different bacterial isolates grown in M9 minimal salts medium (Sigma) supplemented with either succinate, or the auxin inhibitors L-kynurenine, or NPA as carbon sources. For this experiment we used three independent biological replicates.

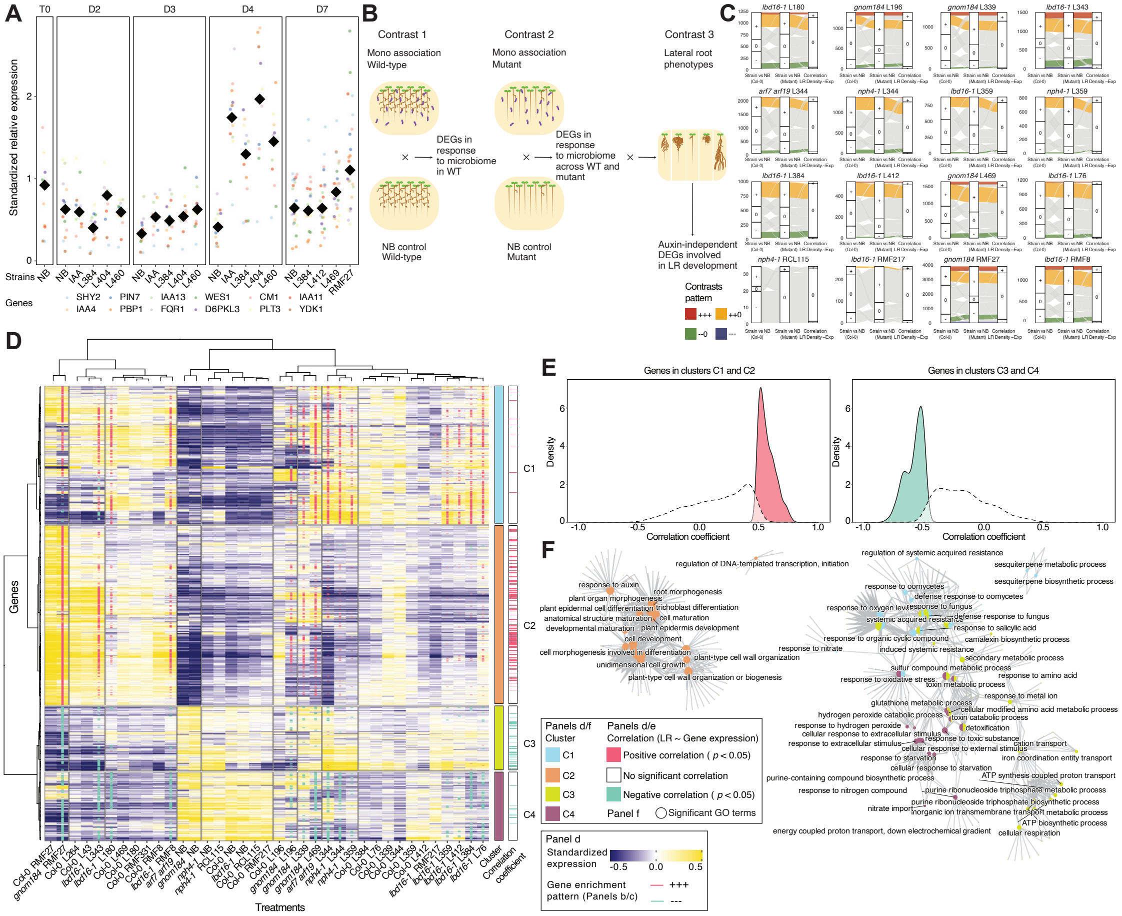

SI Appendix*, Fig. S7. A hierarchical gene filtering strategy on RNA sequencing identified the induction of an ethylene response during lateral root formation in plant colonized by microbes. (*A*).** The graph shows the results of a real-time PCR analysis of the expression of auxin reporter genes, extracted from the literature (*2*, *8*–*12*) in roots of Col-0 wild-type plants exposed to different bacterial isolates. For the analysis, plants were germinated under axenic conditions for a week and then transferred to MS agar plates inoculated with different bacterial strains or to agar plates supplemented with 100 nM auxin (IAA) that we used as a control. Samples were collected at different days (D) post-transferring from the starting point to day 7. Black diamonds in the figure represent the average of the standardized relative expression of the auxin reporter genes in a given treatment and time point. Colors represent different genes analyzed. **(*B*).** Schematic representation of the hierarchical gene filtering strategy used to identify differentially expressed genes involved in lateral root regulation in plants colonized by microbes. Differentially expressed genes (DEGs) in roots of Col-0 wild-type plants grown under axenic conditions were compared to those found in plants exposed to individual bacteria (Contrast 1). In each case, the resulting selection was then filtered by comparing them with those DEGs found to be significantly expressed in lateral root mutants colonized by individual bacteria (Contrast 2). Finally, the resulting gene selection was correlated with the lateral root phenotypes the bacteria produced to identify a set of genes the expression of which correlated with lateral root development (Contrast 3). **(*C*).** Alluvial diagrams showing changes in the number of DEGs after every step of the filtering process described in ***B. Individual diagrams represent the filtering process for the transcriptional response to individual bacterial isolates used. In the plots Y axis represent the number of DEGs and

the vertical bars denoted the filtering steps described in ***B***. The name of the lateral root mutant used in each case is denote in the plots. Gene induction, repression, or no changes in gene expression are represented in the plots with a +, -, 0, respectively. The same nomenclature was used for the last filtering step to indicate a positive (+), negative (-), or no correlation (0) with the lateral root phenotypes. The colored gene flows are those selected for further analysis. **(*D*).** Heatmap of the 2122 DEGs selected after the first two steps of the hierarchical gene filtering strategy (before the correlation with the lateral root density phenotype (contrast 3 in panel ***B***). Vertical clusters are designated with the plant genotype and the bacterial treatments and horizontal clusters with a colored bar (cluster, C) on the *Right*. Small horizontal red and green lines within the heatmap represent genes with a significant different expression. The second vertical bar on the *Right* (Correlation) represents positive (red) or negative (light blue) correlation between gene expression and the lateral root phenotypes. The final selection of DEGs is shown in Fig. 4*A*. **(E).** Distribution of the Pearson correlation coefficient (R) from a correlation analysis between the clusters of DEGs identified in panel ***D*** upregulated (C1 and C2) or downregulated (C3 and C4) and the lateral root phenotypes. Note that this is the last step of the hierarchical gene filtering strategy used here (contrast 3 panel ***B***). In each case, only one of the clusters up- or down-regulated compared showed a high correlation with the lateral root phenotypes and they are colored in red (C2) and light green (C3) in the figure. These two highly correlated clusters were used to build the heatmap of Fig. 4*A*. **(*F*).** Networks of statistically significant gene ontology terms within the different DEGs identified in panel ***D*.** In the figure, network nodes are colored following the cluster's color code defined in ***D***. Networks shows only significant ontologies (hypergeometric test) and the node size denotes the number of genes connected to that particular term. For the RNA-seq experiment we used three independent biological replicates per condition with at least 10 roots each. This experiment was repeated twice (n = 6).

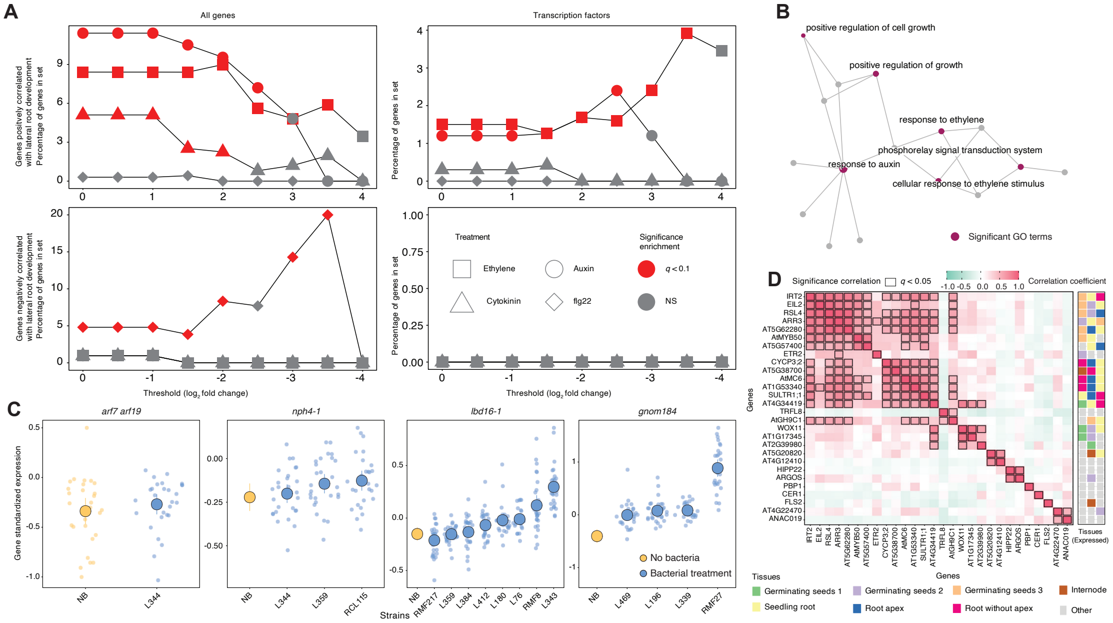

***SI Appendix*, Fig. S8. Members of the root microbiota induce the expression of ethylene-responsive genes during lateral root formation. (*A*)**. Gene set analysis showing the distribution of genes (*Left*) and transcription factors (*Right*) from the phytohormones (Ethylene (*4*), Auxin (*8–12*), Cytokinin (*13*) and the PAMP flg22 (*14*)) core genes sets identified from literature, across the genes that positively (*Top* panel) or negatively (*Bottom* panel) correlated with lateral root phenotypes identified in Fig. 4*A* and ordered by level of expression (log2 fold change). Red color indicates statistical significance (hypergeometric test, $q < 0.1$). **(*B*).** Network of statistically significant gene ontology terms within the 28 DEGs identified in response to bacterial isolates that overlap with the ethylene core genes from the literature (*4*). In the figure, the network shows only significant ontologies (hypergeometric test) and the colored nodes represent overrepresented terms. **(*C*).** Level of induction (standardized expression) of the identified 28 ethylene-related genes in the lateral root mutants *arf7 arf19, nph4-1, ldb16-1, gnom184* in response or not (NB, yellow) to different bacterial isolates (blue). **(*D*).** Heat map showing the pairwise correlation in expression of the 28 ethylene responsive genes induced in response to the member of the plant microbiota during lateral formation across tissues found in the Klepikova Atlas (*15*). At least, fourteen of the 28 genes are highly correlated across the Atlas. The bar on the *Right* represents the tissues where the genes are expressed according to the Atlas and the different colors represent different tissues. Statistical significance of the overlap of enriched genes was determined via hypergeometric test and significant values are highlighted with a black square ($q < 0.05$). For the RNA-seq experiment we used three independent biological replicates per condition with at least 10 roots each. This experiment was repeated twice (n = 6).

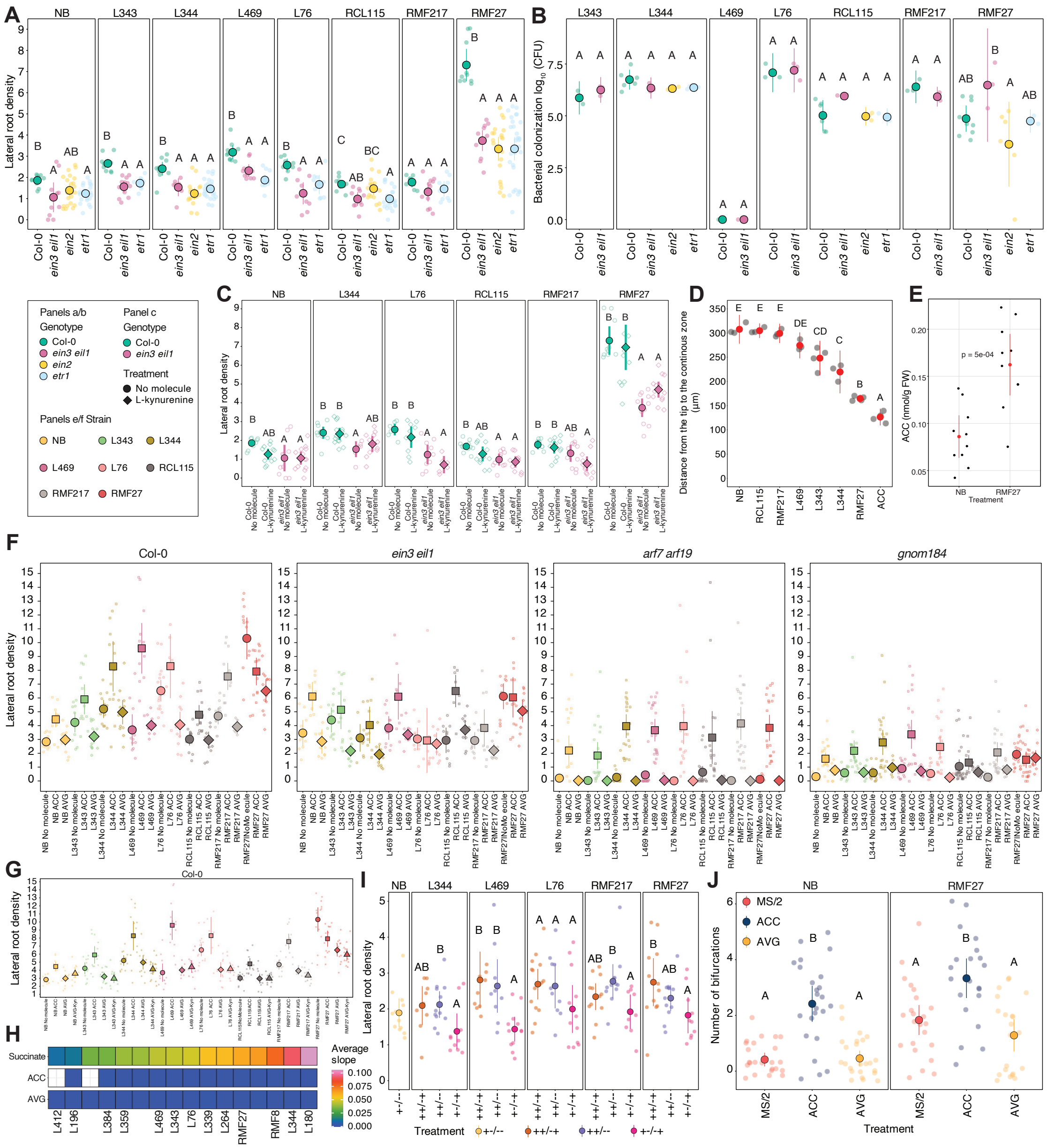

***SI Appendix,* Fig. S9. Ethylene signaling regulates lateral root development in response to members of the plant microbiota. (*A*).** The graph shows the lateral root density in wild-type plants and ethylene mutants *ein3 eil1*, *ein2*, and *etr1* exposed or not (NB) to individual bacterial isolates. Different colors represent different plant genotypes. The names of the bacterial isolates used are on *Top* of each panel. Significance was determined via linear modelling, letters represent the compact letter display of a Tukey post-hoc test. This dataset was used in Fig. 5*A*. **(*B*).** The graph shows the colonization capacity of different bacterial isolates determined in roots of wild-type plants and the ethylene mutants *ein3 eil1, ein2, and etr1* using log-transformed-colony forming units. Colors represent the different genotypes used. Significance was determined via linear modelling, letters represent the compact letter display of a Tukey post-hoc test. For this experiment we used two independent biological replicates per treatment with at least 10 plants each. We repeated this experiment twice. **(*C*).** Graph showing the lateral root

density in wild-type plants and the ethylene mutant *ein3 eil1* colonized with different bacterial isolates in the presence or not (No Molecule) of the auxin biosynthesis inhibitor L-Kyn. As a control we used wild-type plants and the ethylene mutant *ein3 eil1* grown axenically (NB) in the presence or not (No Molecule) of L-Kyn. Colors represent different genotypes. Significance was determined via linear modelling, letters represent the compact letter display of a Tukey post-hoc test. For this experiment we used two independent biological replicates per treatment with at least 10 plants each. We repeated this experiment twice. **(*D*).** Quantification of the β-Glucuronidase (GUS) signal in the ethylene sensor *EBS:GUS*, that express the GUS reporter under the control of a synthetic *EIN-3*-responsive promoter. As a proxy for GUS activity, we quantified the distance from the root tip to the continuous area of GUS activity in the root of *EBS:GUS* plants exposed or not (NB) to different bacteria isolates. As a control we used *EBS:GUS* plants treated with the ethylene precursor 1-Aminocyclopropane-1-carboxylate (ACC). Significance was determined via linear modelling, letters represent the compact letter display of a Tukey post-hoc test. Note that an increase in GUS activity reduces the distance from the root tip to the zone of continuous detection of GUS activity. We measured at least 3 plants per condition. **(*E*)** The graph shows the normalized amount (nmol/gFW) of the ethylene precursor ACC found in the root of wild-type plants exposed or not (NB) to the bacterial isolate RMF27. The *p*-value resulting from a *t*-test comparing both samples is shown in the figure. We used five independent biological replicates per treatment with at least 100 roots each. We repeated this experiment twice (n = 10). **(*F*)** The graph shows the lateral root density in wild-type plants, the ethylene mutant *ein3 eil1* and the lateral root mutants *arf7 arf19* and *gnom184* inoculated with different bacterial isolates, and treated or not (No molecule) with the ethylene precursor ACC, or with the ethylene biosynthesis inhibitor AVG. As a control we used wild-type plants, the ethylene mutant *ein3 eil1*, and the lateral root mutants *arf7 arf19* and *gnom184* grown axenically (NB) treated or not (No molecule) with ACC or AVG. Colors represent different bacterial isolates and shapes represent the chemical treatments. **(*G*).** The graph shows the lateral root density in wild-type plants inoculated with different bacterial isolates, and treated or not (No molecule) with the ethylene precursor ACC, with the ethylene biosynthesis inhibitor AVG, or with the ethylene biosynthesis inhibitor AVG combined with the auxin biosynthesis inhibitor L-Kyn. As a control we used wild-type plants grown axenically (NB) treated or not (No molecule) with ACC, AVG, or with AVG combined with L-Kyn. Colors represent different bacterial isolates and geometrical shapes represent the chemical treatments used. Note the lack of additive effect between the AVG and L-Kyn treatments. We used two independent biological replicates per treatment with at least 10 plants each. We repeated this experiment twice. **(*H*).** The heatmap shows the slope of the exponential phase of the growth curves of different bacterial isolates grown in M9 minimal salts medium (Sigma) supplemented with either succinate, ACC, or AVG as carbon sources. We used three independent biological replicates. **(*I*).** Graph showing the lateral root density of wild-type plants grown on split plates that have two compartments physically separated by a plastic barrier that prevents the media contact but not the gas exchange between compartments. For each bacterium used we designed four treatments: (plant + no bacteria) *vs* (no plant + no bacteria) [+ -/- -]; (plant + bacteria) *vs* (no plant + bacteria) [+ +/- +]; (plant + bacteria) *vs* (no plant + no bacteria) [+ +/- -]; (plant + no bacteria) *vs* (no plant + bacteria) [+ -/- +]. In the figure, colors denote different treatments. Significance was determined via linear modelling, letters represent the compact letter display of a Tukey post-hoc test. Notice that only plants colonized by microbes (physically in contact) can change lateral root density, demonstrating that no bacterial volatiles are involved in this mechanism. We used two independent biological replicates per treatment and we repeated it twice (n = 4). **(*J*).** The graph shows the effect of the ethylene precursor ACC or the ethylene biosynthesis

inhibitor AVG on root bifurcation in *Selaginella* explants grown axenically (MS/2) or colonized by the bacterium RMF27. Colors represent different treatments. Significance was determined via linear modelling, letters represent the compact letter display of a Tukey post-hoc test. We used two independent biological replicates with 12 explants each.

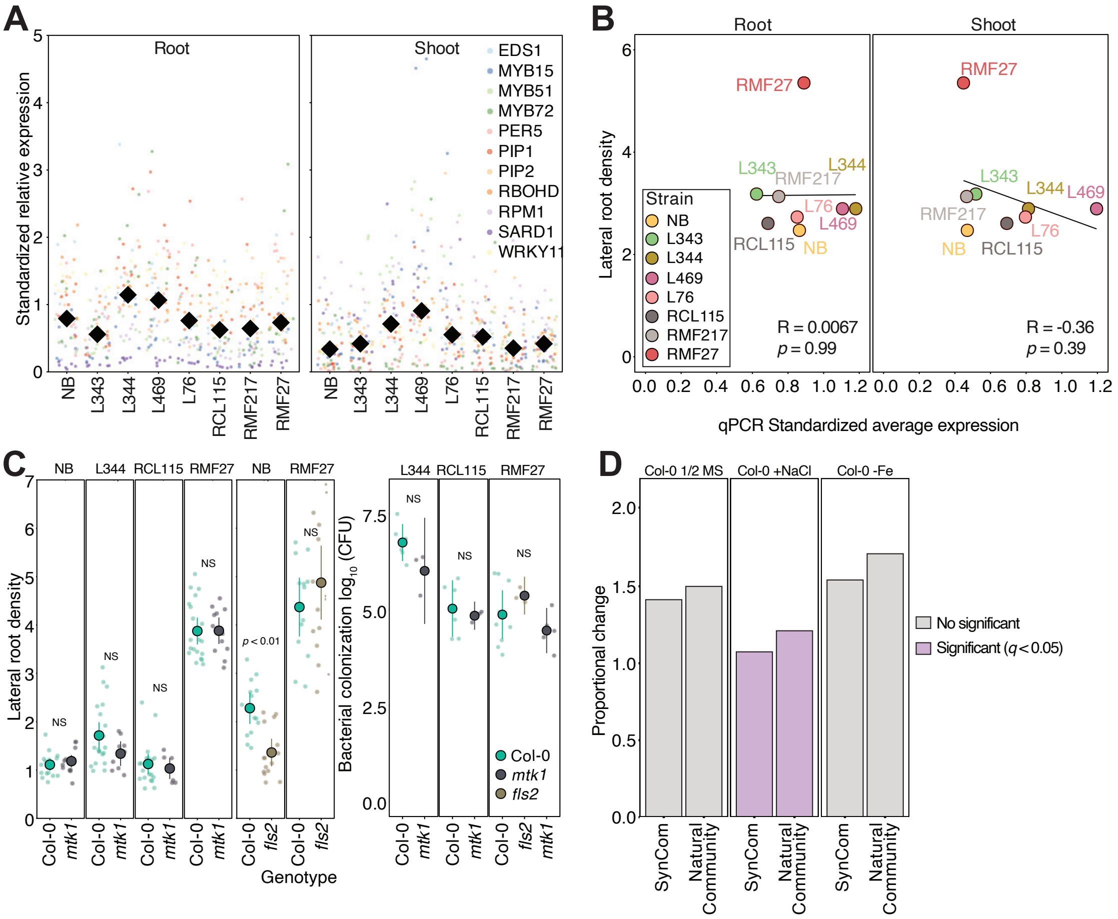

SI Appendix,* Fig. S10. The effect of the microbiota on lateral roots might not require the activation of the plant's immune system. (*A*).** The graph shows the results of a RT-PCR analysis of the expression of defense marker genes (*16*, *17*), in roots (*Left*) and shoots (*Right*) of Col-0 wild-type plants colonized by different bacterial isolates. For the analysis, plants were germinated under axenic conditions for a week and then transferred to the different bacterial treatments. Black diamonds in the figure represent the average of the standardized relative expression of the defense marker genes in a given treatment. Colors denote different genes analyzed. **(*B*).** Scatterplots showing the Pearson correlation analysis between the lateral root density and the standardized expression of defense genes analyzed in panel ***A in roots (*Left*) and shoots (*Right*) of wild-type plants exposed to different bacterial isolates. Colors represent different bacterial isolates. In each case, the correlation coefficient R and its *p*-value are shown. We used three independent biological replicates per treatment with 10 plants each. We repeated this experiment twice (n = 6). **(*C*).** (*Left*) The graph shows the lateral root density in wild-type plants, the PAMP receptor mutant *fls2*, and the ethylene synthesis mutant *mtk1* colonized or not (NB) with different bacterial strains. Colors denote different genotypes used. (*Right*) Graph showing the colonization capacity of the bacteria used determined in roots of wild-type plants Col-0, the ethylene mutant *mtk1*, and the PAMP receptor mutant *fls2* using log-transformed colony forming units (Log_{10}(CFU)). Colors represent different plant genotypes. We used two independent biological replicates per treatment with 10 plants each. We repeated this experiment twice. **(*D*)** Bar graphs showing the proportion of changes in lateral root density values in wild-type Col-0 plants inoculated with a 16-member synthetic community (SynCom) or a natural community relative to axenic plants, in full nutrient condition

(1/2MS) and two abiotic stresses, high salinity (+NaCl) and low iron (-Fe). Bars colored in grey denote not significant differences and purple means significant differences with full nutrient conditions (Dunnet test, $q < 0.05$). For this figure, we used the data from Fig. 5*E* and *F*.

Datasets

Dataset S1. Collection of bacterial strains used in this work. Column A of the dataset (Strain) contains the code used to identify the individual strains. Column B has the numerical identifier (taxon_oid) used to retrieve the bacterial strain genome sequence from https://img.jgi.doe.gov/. From column C to J the dataset describes the taxonomy of the strains. The last columns (K to T) show information about the presence (YES) or Absence (NO) of operons related to auxin synthesis or degradation in the strain genomes.

Dataset S2. Bacterial strains modify plant root architecture. The dataset shows the average value, for each root feature, determined in plants exposed to the different bacterial strains (Strain ID) and the position of the strains in the clusters defined in Fig. 1*D*.

Dataset S3. List of genes identified in our RNA-seq experiment after the hierarchical gene filtering with an AvLog2FoldChange>=3. The dataset also shows gene identifiers, gene names, and other gene characteristics including gene family, gene function, gene description, and gene ontology information.

Dataset S4. Bacterial strains selected for the definition of the microbiota-driven mechanism controlling lateral root development. Column A of the dataset (Strain) contains the code used to identify the individual strains. Column B has the numerical identifier (taxon_oid) used to retrieve the bacterial strain genome sequence from https://img.jgi.doe.gov/. From column C to J the dataset describes the taxonomy of the strains. Columns K and L show information about the presence (YES) or Absence (NO) of operons related to auxin synthesis or degradation in the strain genomes. From column M to O the dataset shows information about the flg22 coding genes present in the strain genomes. Columns P and Q show information about the presence (YES) or Absence (NO) of operons related to ethylene synthesis in the bacterial genomes.

Dataset S5. Direct quantification of 1-Aminocyclopropane-1-carboxylate (ACC) in plant roots. The dataset shows the normalized amount of ACC detected in roots of Col-0 wild-type plants exposed or not (NB) to the bacterial isolate RMF27. This data was used to create the *SI Appendix*, Fig. S9*E*.

Dataset S6. *Arabidopsis thaliana* mutants and lines used in this work. See Methods section. The dataset shows the names of the mutants and lines used in this work, the mutant ID, the name and affiliation of the seed providers, and the literature reference where these mutants and lines have been published.

Dataset S7. RT-PCR primers used in this work. See materials and methods section. The dataset shows the name of the genes, their gene ID, the sequence of the primers used in the RT-PCR analyses to amplify these genes, the methods section where the experiment is described, and the number of the figure where the results are shown.

Materials and Methods

1. Screen of bacterial isolates based on their ability to change root architecture.

a. Growth conditions for the bacterial strains and plant inoculation.

The bacterial isolates used here belong to two collections of bacterial strains: one isolated from roots and one isolated from leaves. The strains from the root collection were isolated from roots of healthy Brassicaceae (mostly *Arabidopsis thaliana* (*Arabidopsis*)) grown in two natural soils from North Carolina, USA (*18*). For the leaf collection, bacteria were isolated from leaves of healthy *Arabidopsis* plants collected from six locations around Tübingen, Germany, and Zurich, Switzerland (*19*).

All experiments started from single bacterial colonies. To isolate the colonies, -80°C glycerol bacterial stocks were grown on LB plates in the case of the root collection and on R2A plates for the leaf collection. The composition of the R2A was: Casein acid hydrolysate 0.5 g/L, Yeast extract 0.5 g/L, Protease peptone 0.5 g/L, Dextrose 0.5 g/L, Starch 0.5 g/L, Dipotassium phosphate 0.3 g/L, Magnesium sulfate 0.024 g/L, Sodium pyruvate 0.3 g/L, agar 15g/L, pH 7.2 supplemented with Methanol 0.5%).

All plates were incubated at 28°C until it was possible to visualize the colonies. For some slow growers, this process took several days.

Then, a single colony was inoculated into sterile 15 mL falcon tubes containing approximately 4 mL of LB medium or R2A medium supplemented with 0.5% Methanol for the root and leaf collection, respectively. Tubes were placed in an incubator and the cultures were grown at 28°C with agitation at 250 r.p.m. To wash bacterial cells, all cultures were centrifuged in a benchtop centrifuge (Eppendorf 5810R) at 3220 g at 4°C, and the pellets were washed using 10 mM $MgCl_2$. This step was repeated three times. In the last

step, bacterial pellets containing washed cells were resuspended in 1 mL of 10 mM $MgCl_2$ using a vortex. To equalize the number of cells per culture, the OD_{600nm} was measured and assuming that 1 OD_{600nm} unit is equal to 10^9 c.f.u/mL individual bacterial inocula were prepared at a final concentration of 10^5 c.f.u/mL. The individual cultures (100 µL) were spread on the surface of 12 X 12 cm square agar plates using an L-shaped cell spreader (Fisher scientific) before transferring the seedlings. We used two independent replicates per bacterial condition.

b. Seed sterilization.

All *Arabidopsis* Col-0 seeds were sterilized using a solution of 70% ethanol and 0.05% Triton X-100 (Sigma) for 15 minutes with agitation. Then, seeds were washed with sterile distilled water at least three times to eliminate the ethanol. Seeds were stratified in the dark at 4°C for at least 24 hours before use.

c. In vitro plant growth conditions.

To study the function of members of the plant microbiota in the regulation of lateral root development, we analyzed the effect of the 391 individual bacterial isolates from the root and leaf collections on plant root architecture. This selection of bacteria covers most of the phylogenetic diversity found in the microbiome colonizing *Arabidopsis* in nature (*4*).

Sterilized Col-0 seeds were first germinated on agar plates containing 0.5x MS (Murashige and Skoog, Sigma) medium solidified with 1% bacto-agar (Sigma) for a week. Then, approximately ten seedlings with similar development were transferred to a new 0.5x MS agar plate inoculated with the individual isolates. As a control we also transferred ten seedlings to an uninoculated plate with only 10 mM $MgCl_2$ with no bacteria present (No-bacteria control). Plates were manually randomized and placed in a growth chamber and

grown in a vertical position under a 16-h light/8-h dark regime at 21°C day/19°C night for another 7 d. We used two independent replicates per bacterial condition.

d. Determination of root architecture parameters.

We quantified twenty-one features related with root architecture in *Arabidopsis* Col-0 exposed or not to individual bacterial isolates from pictures, using the SmartRoot software (*20*). The root features analyzed were percentage of the primary root covered by lateral roots (LR coverage), lateral roots density (LR density), total lateral roots volume (LR volume), total lateral root surface (LR surface), lateral root length (LR length), distribution of the lateral root length (LR length distribution), angle formed by the lateral roots and the primary root (LR angle), diameter of the lateral root (LR diameter), number of lateral roots in each side of the primary root (LR symmetry), distance between two consecutives lateral roots (LR interbranch distance), distance from the root tip to the first lateral root (Distance to the 1st LR), number of lateral roots with secondary lateral roots (Number of branched LR), number of secondary lateral roots (2ndLR number), diameter of the secondary lateral roots (2ndLR diameter), length of the secondary lateral roots (2ndLR length), total volume of the secondary lateral roots (2ndLR volume), total surface of the secondary lateral roots (2ndLR surface), diameter of the primary root (PR diameter), volume of the primary root (PR volume), surface of the primary root (PR surface), and primary root length (PR length).

e. Computational and statistical analysis of the data.

The phylogenetic tree of the screened bacterial isolates was built using the supermatrix approach described in Levy et al. (*18*). Thirty-one marker genes were scanned across the bacterial genomes in our collections using the hmmsearch tool from the hmmer version 3.1b2 (*21*). Next, we aligned each individual marker using MAFFT (*22*), and the low-quality columns in the alignment were filtered using trimAl (*23*). Then, all filtered alignments were

concatenated into a super alignment and the phylogeny was inferred with FastTree version 2.1 (*24*) using the WAG model of evolution. The phylogenetic tree was visualize using the iTOL interface (*25*).

We used the phylogenetic tree and the phytools v0.6-99 (*26*), R package (*27*), to determine the phylogenetic signal (Pagel's Lambda) for the individual root architecture markers quantified across all isolates in the dataset.

To determine the total number of bacterial strains modifying individual root architecture features, we normalized each of the 21 root features using the no bacteria (NB) treatment present in all the batches for which the 391 distinct isolates were split into. To do so, we estimated a batch normalization factor (one per feature) by dividing the average measurement across the NB samples of that batch over the computed average measurement in all the NB samples across all batches. Finally, we normalized all measurements in a given batch by multiplying each one of them by their corresponding estimated normalization factor. In order to reduce the skewness within each feature and to be able to compare magnitudes across features we transformed the batch-corrected measurements by applying a $\log_{10}$ transformation followed by a z-score standardization of each feature.

For each of the 391 distinct isolates, we compared each of the 21 root features against the no bacteria (NB) treatments within the corresponding strain batch. To do so, we applied the "two sided" Wilcoxon signed-ranked test, implemented in the wilcox.test function from the stats R package (*27*), taking the standardized value of each feature. We used the false discovery rate (FDR) method, implemented in the p.adjust function from the

stats R package (*27*), to adjust the *p*-values obtained for all comparisons. Comparisons with FDR adjusted *p*-value < 0.05 were considered significant.

For each of the 21 quantified root features, we computed the coefficient of variation within each feature by taking the $\log_{10}$ transformed measurements and applying the following formula

$$CV = \frac{\sigma}{\mu}$$

σ = standard deviation of each feature across the 391 isolates

μ = mean of each feature across the 391 isolates

To determine the correlation among features, we constructed a strain-by-feature matrix by computing the average value ($\log_{10}$ and standardized) of each of the 21 root features for each of the 391 isolates used. Next, we input the aforementioned matrix to calculate the all-*vs*-all Pearson correlation coefficients between the 21 root features. We subjected this correlation dissimilarity matrix to hierarchical clustering using the ward.D method implemented in the rcorr function from the Hmisc v4.6.0 R package (*27*). Finally, asymptotic *p*-values were false discovery rate (FDR) corrected and reported for each of all *vs* all feature comparisons.

For cluster analysis of root features, we used the chibi.heatmap function from the ohchibi R package (*27*) to apply hierarchical clustering (ward.D method) over a dissimilarity matrix (Euclidean distance) calculated from the matrix describing for each strain the estimate of each feature against the no bacteria (NB) treatment. Clusters of strains with similar root features pattern were delimited via dendrogram cutting through the function cutree from the stats R package (*27*).

To determine the association between the bacterial effect on primary and lateral roots, we compared the distinct patterns of change between primary root length and lateral root density with respect to the no bacteria (NB) treatment by plotting in a scatterplot the obtained estimates of both features for each of the 391 isolates assayed. The Pearson correlation coefficient between both features was computed and displayed via the stat_cor function from the ggpubr package v.0.4.0.

Finally, we compared the distributions of the 391 estimates for the primary root and lateral root density quantifications using the Kolmogorov-Smirnov test coded in the function ks.test from the stats package of R (*27*).

For the identification of auxin related operons in the bacterial genomes, we employed the literature-curated biosynthetic and degrading pathways described in Metacyc (*28*) to identify Clusters of Orthologous Genes (COG) and Kyoto Encyclopedia of Genes and Genomes (KEGG) orthology identifiers of key enzymes involved in the biosynthesis and degradation of auxin. Delineation of these functional orthology IDs across the 373 sequenced isolates used in this screening was based on the IMG-based functional annotation of each genome (*29*). To delineate each pathway, the following functional markers were used:

For the auxin biosynthesis III bacterial pathway, we utilized the enzyme E.C:1.13.12.3, tryptophan 2-monooxygenase (KEGG id: K00466) as pathway marker.

For the auxin biosynthesis IV bacterial pathway, we utilized the enzyme EC:4.2.1.84, nitrile hydratase subunit alpha and beta (KEGG id: K01721) as pathway marker.

For the auxin biosynthesis V bacterial pathway, we utilized the enzyme EC 3.5.5.1, nitrilase (KEGG id: K01501) as pathway marker.

For the auxin biosynthesis VI bacterial pathway, we utilized the enzyme EC: 4.1.1.74, indolepyruvate decarboxylase (KEGG id: K04103) as pathway marker.

For auxin degradation, we employed the HMM-based pipeline (*30*) that *de novo* identifies *iac*-like and *iad*-like auxin degradation operons across genome assemblies. Isolates for which there was no genomic evidence of auxin biosynthetic or degrading capabilities were labelled as “IAA operon absence”. The remaining of the isolates were labelled as “IAA operon presence”.

We compared the effect of isolates, harboring or lacking auxin biosynthetic and degradation capabilities, over the root architecture features utilizing two approaches. The first approach compared, for each feature, the distribution of estimates (with respect to the no bacteria (NB) treatment) of both type of isolates (operon presence *vs* absence). Differences between the distributions of effects across types of isolates were determined via the Kolmogorov-Smirnov test coded in the function ks.test from the stats package of R (*27*).

The second approach compared for each cluster of root architecture features (Fig. 1*D*) the within cluster pattern of isolates harboring or lacking auxin biosynthetic and degradation capabilities, via the adonis function from the R package vegan v.2.5.7 (*27*).

For the identification of ethylene biosynthesis II bacterial pathway, we utilized all the homologous of the enzyme 2-oxoglutarate dioxygenase (ethene-forming enzyme; EFE) E.C: 1.13.12.19 reported in KEGG. We aligned all the homologous enzyme sequences using MAFFT (*31*) and a profile HMM (hidden Markov models) was generated. All bacterial genomes were scanned to detect the presence of the profile HMM. A coverage of 70% of the original profile HMM was considered as a criterium to define the presence of the enzymes in the bacterial genomes.

For the ethylene biosynthesis III bacterial pathway, we utilized the enzyme methionine transaminase EC: 2.6.1.88 (KEGG id: K14287) as pathway marker.

2. Analysis of the individual bacterial effects on *Selaginella moellendorffii* and *Arabidopsis* lateral root mutants.

a. *Growth conditions for the bacterial strains and plant inoculation.*

The 99 selected bacteria, spanning across all clusters identified in Fig. 1*D*, were inoculated from glycerol stocks into 15-mL sterile falcon tubes containing 4 mL of LB medium, and grown at 28°C, with agitation at 250 r.p.m. To wash bacterial cells from spent medium and cell debris all individual cultures were centrifuged in a benchtop centrifuge (Eppendorf 5810R), 3220 g at 4°C, and washed three times with 10 mM $MgCl_2$. Clean pellets were resuspended in 1 mL of 10 mM $MgCl_2$, and the OD_{600nm} was measured. We equalized individual bacterial strain concentrations to 10^5 c.f.u/mL assuming that 1 OD_{600nm} unit is equal to 10^9 c.f.u/mL. Individual bacterial inocula (100 µL) were spread using a L-shaped cell spreader (Fisher scientific) onto the surface of 12 X 12cm square agar plates (Greiner Bio-One) containing 0.5x MS, 0.8% bacto-agar (Sigma) medium before transferring the explants.

b. In vitro plant growth conditions.

Selaginella moellendorffii (*Selaginella*) plants were grown axenically from explants in closed magenta jars containing 0.5x MS (Sigma) medium solidified with 0.4% bacto-agar (Sigma) under a 16-h light/8-h dark regime at 21°C day/19°C night for two months, sufficient time to generate fully developed *Selaginella* plants. New *Selaginella* explants were prepared from the magenta cultures using scalpel and forceps under sterile conditions and approximately 12 explants of similar sizes lacking roots were then transferred to each of the individual-strain inoculated plates with 0.5x MS, 0.8% agar. As a control we also transferred *Selaginella* to no-bacteria (NB) treatments, plates inoculated with 100 µL of 10 mM $MgCl_2$. Plates were randomized and placed in a growth chamber and grown in a vertical position under a 16-h light/8-h dark regime at 21°C day/19°C night for 14 d. For each condition, plates were prepared in duplicates.

To determine the influence of members of the microbiota on lateral root development, we analyzed a collection of *Arabidopsis* mutants (*iaa14 slr-1*, *arf7 arf19*, *nph4-1*, *lbd16-1, gnom184*, *gelpquint1*, and *gelp72*), and the line *pCASP1::CDEF1* that express the cuticle destructing factor1 (CDEF1) that degrades suberin (Dataset S6). These genotypes represent different levels of impairment in lateral root formation and emergence. All these mutants and the line were in the Columbia (Col-0) background. Mutant seeds were surface sterilized and germinated on agar plates containing 0.5x MS (Sigma) medium solidified with 1% bacto-agar (Sigma) for seven days. Approximately ten seedlings of each mutant were transferred to each of the individual-strain inoculated agar plates containing 0.5x MS, and to the no-bacteria (NB) control plates inoculated with 100 µL of 10 mM $MgCl_2$. Plates were manually randomized and placed in a growth chamber and grown in a vertical position under a 16-h light/8-h dark regime at 21°C day/19°C night for another 7 d. To

confirm the observed root phenotypes some plates were grown for 14 d. All mutants were analyzed at least twice during the course of the experiments.

To evaluate the effect of nutrient deficiency on lateral root development, we sterilized and germinated seeds of Col-0 and lateral root mutants (*arf7 arf19*, *nph4-1*, *lbd16-1, gnom184*) (Dataset S6) on agar plate containing 0.5x MS (Sigma) medium solidified with 1% bacto-agar (Sigma) for 7 d. Approximately ten seedlings of each mutant and Col-0 plants were transferred to new agar plates containing 0.5x MS, 0.005x MS, and 0.0005x MS solidified with 1% bacto-agar and inoculated with 100 µL of 10 mM $MgCl_2$. Plates were manually randomized, and placed in a growth chamber and grown in a vertical position under a 16-h light/8-h dark regime at 21°C day/19°C night for another 7 d. We used five independent replicates per condition.

To study how the microbiota controls lateral root formation we sterilized and grew Col-0 wild-type plants, the lateral root primordia marker lines *pSKP2B::GUS* line (*6*) and *pLBD16:GFP* (*7*), the pre-branch site marker line *DR5:Luciferase* (*2*), and the auxin sensor *DII:VENUS* (*32*) seeds on agar plates with 0.5x MS (Sigma) medium inoculated with 100 µL of 10 mM $MgCl_2$ (NB control) or the individual bacterial isolates. Plates were manually randomized and placed in a growth chamber and grown in a vertical position under a 16-h light/8-h dark regime at 21°C day/19°C night for 6 d. We used two independent replicates.

To determine the effect of auxin signaling and transport on lateral root development we also sterilized and germinated Col-0 wild-type seeds on agar plates with 0.5x MS, 1% bacto-agar (Sigma) for seven days. Then, approximately ten seedlings of Col-0 plants were transferred to new agar plates containing 0.5x MS (Sigma), supplemented with 5 µM

N-1-naphthylphthalamic acid (NPA, an inhibitor of auxin polar transport, (Sigma)) or 1.5 µM L-kynurenine (L-Kyn, an inhibitor of auxin biosynthesis, (Sigma)) solidified with 1% bacto-agar and inoculated or not with the bacterial isolates. Plates were manually randomized, placed in a growth chamber and grown in a vertical position under a 16-h light/8-h dark regime at 21°C day/19°C night for another 7 d. We used two independent replicates and the selected conditions were repeated at least twice.

c. Determination of branching parameters

The number of bifurcation events in *Selaginella* in response to members of the plant microbiota was determined directly from the plates using a dissecting microscope. We considered that a root was bifurcated when a clear Y was formed at the extreme of the roots.

We determined the primary root length and the number of secondary roots in wild-type plants and the lateral root mutants from images taken using a linear robot camera. Primary root length and the number of secondary roots were measured using Image J (*33*) and SmartRoot software (*20*). The density of lateral roots was calculated dividing the number of secondary roots by the length of the primary root.

d. Mineral nutrient analysis

The concentration of mineral nutrient analysis in plant shoots was determined using Inductively Coupled Plasma Mass Spectrometry (ICP-MS). For this analysis, the entire rosette was collected from ten 14-d-old plants grown on agar plates (approximately 0.2 g of fresh weight) using plastic forceps and a ceramic scalpel. Rosettes were sequentially washed three times with 18.2 MΩcm Milli-Q Direct water (Merck Millipore) to remove rest of agar in plastic beakers. After washing, samples were placed in Pyrex digestion tubes

and dried at 88˚C for 20h. After cooling, eight of approximately 108 samples from each sample set were weighed on Mettler five-decimal analytical balance. Then, 1 mL of concentrated trace metal grade nitric acid Primar Plus (Fisher Chemicals) was added to each tube to digest the samples. The nitric acid used was previously spiked with 20 µg/L of Indium that was used as an internal standard for assessing errors in dilution, variations in sample introduction in the equipment, and plasma stability in the ICP-MS instrument. The digestion of the samples was performed in DigiPREP MS dry block heaters (SCP Science; QMX Laboratories) for at least 4h at 115˚C. The digested material was then diluted to 10 mL with 18.2 MΩcm Milli-Q Direct water and the elemental analysis was performed using an ICP-MS, PerkinElmer NexION 2000 equipped with Elemental Scientific Inc 4DXX FAST Dual Rinse autosampler, FAST valve and peristaltic pump. We monitored twenty-four elements that included the stable isotopes: ^{7}Li, ^{11}B, ^{23}Na, ^{24}Mg, ^{31}P, ^{34}S, ^{39}K, ^{43}Ca, ^{48}Ti, ^{52}Cr, ^{55}Mn, ^{56}Fe, ^{59}Co, ^{60}Ni, ^{63}Cu, ^{66}Zn, ^{75}As, ^{82}Se, ^{85}Rb, ^{88}Sr, ^{98}Mo, ^{111}Cd, ^{208}Pb and ^{115}In. As a collision gas we used Helium gas at a flow rate of 4.5 mL/min. To exclude possible polyatomic interferences, we used Kinetic Energy Discrimination mode (KED) to measure Na, Mg, P, S, K, Ca, Ti, Cr, Mn, Fe, Ni, Cu, Zn, As, Se, and Pb and the standard mode for the rest of the elements. Any isobaric interferences were automatically corrected by the instrument Syngistix™ software for ICP-MS v.2.3 (Perkin Elmer). The ICP-MS measurements were performed in peak hopping scan mode with dwell times ranging from 25 to 50 ms depending on the element, 20 sweeps per reading and 3 replicates. The ICP-MS conditions were as follow: RF power – 1600 Watts, auxiliary gas flow rate 1.20 L/min. Torch alignment, nebulizer gas flow and quadrupole ion deflector (QID) voltages were optimized before analysis for highest intensities and lowest interferences with NexION Setup Solution containing 1 µg/L of Be, Ce, Fe, In, Li, Mg, Pb, and U in 1% nitric acid using a standard built-in software procedure. We used liquid reference material that was prepared using pooled digested samples to correct for

variation between and within ICP-MS analysis runs. This reference material was run after the instrument calibration, and also after every nine samples in all ICP-MS sample sets.

Sample concentrations were calculated using external calibration methods within the instrument software. Further data processing including calculation of final elements concentrations was performed in Microsoft Excel. First, sample sets that have been run at different times were connected as an extension of the single-run drift correction. Linear interpolation between each pair of liquid reference material standards was used to generate a theoretical standard for each sample that was then used to correct the drift by simple proportion to the first liquid reference material standard analyzed in the first run. Second, the blank concentrations were subtracted from the sample concentrations and then the final element concentrations were obtained by multiplying by the dilution factor and normalizing the element concentrations to the samples calculated dry weight. To increase the throughput of the protocol, we avoided the time-consuming process of weighing each sample on a balance. For every 108 samples the dry weights of eight reference samples were measured and used to calculate the weights and then final element concentration of the remaining samples based on a heuristic algorithm which uses the best-measure elements in these samples, the weights of the eight weighed samples and the solution concentrations as described in Lahner et al. (*34*).

e. In vitro auxin synthesis and degradation assays

To confirm that the selected bacterial strains are not producing or degrading auxin in in vitro conditions we grew the bacterial isolates in 96 well plates in triplicate for two days at 28°C with agitation at 250 r.p.m. This time was sufficient to reach the stationary phase of all cultures used.

For IAA synthesis we used a modified method from (*35*). IAA production was detected in bacterial culture grown in 200 µL of LB medium enriched with 500 $\mu g \cdot L^{-1}$ L-tryptophan (Sigma).

In each culture IAA were determined using a modified version of the Salkowski method (*36*). For each sample, 100 µL of Salkowski reagent (10 mM $FeCl_3$ in 35% perchloric acid) was mixed with 50 µL of culture supernatant and incubated for 30 min in dark. The presence of IAA was determined by measuring the absorbance at 530 nm using a FLUOstar® Omega (BMG LABTECH). As a control of the method, we mixed also Salkowski reagent with 50 µL of 40 mM IAA. As a control we used a culture of *Escherichia coli* DH5α.

For the IAA degradation assays, bacterial isolates were grown in M9 minimal salts medium (Sigma) supplemented with 2 mM $MgS0_4$, 0.1 mM $CaCl_2$, 10 µM $FeSO_4$, and either 0.4 mM IAA or 15 mM succinate (Sigma) as carbon sources. All cultures were grown for two days at 28°C with agitation (250 r.p.m). This time was sufficient to reach the stationary phase of all cultures used. In all cases, the bacterial growth was monitored by measuring the OD at 600nm. IAA was determined using a modified version of the Salkowski method (*36*). For each sample, 100 µL of Salkowski reagent (10 mM $FeCl_3$ in 35% perchloric acid) was mixed with 50 µL of culture supernatant and incubated for 30 min in dark. The presence of IAA in the bacterial cultures was determined by measuring the absorbance at 530nm using a FLUOstar® Omega (BMG LABTECH). As a control of the method, we mixed also Salkowski reagent with 50 µL of M9 growing solution. As a negative control we used a culture of *E. coli* DH5α that does not use IAA as a carbon source.

f. Lateral root primordia quantification

To visualize and quantify the number of lateral root primordia at the different developmental stages, we used 6-d-old Col-0 wild-type plants and lateral root mutants, *arf7 arf19*, *nph4-1*, *lbd16-1, gnom184*, direct germinated in axenic conditions or in contact with the selected bacterial isolates. To quantify the lateral root primordia, roots were fixed in 20% methanol and 4% hydrochloric acid for 15 min. Then, roots were cleared using a solution of 60% ethanol in 7% NaOH for 15 min, followed by successive soaks in 40% ethanol, 20% ethanol, 10% ethanol for 5 min. Samples were stored in 50% glycerol until use. For the analysis, roots were placed on microscope slides and covered with a coverslip. Lateral root primordia in the entire root were quantified using a Leica 2 DM5000B fluorescence microscope at 40x magnification.

g. Detection of β-Glucuronidase activity

To quantify the number of lateral root primordia we also used the line *pSKP2B::GUS* (Dataset S6). This line expressed the β-Glucuronidase (GUS) enzyme under the promoter of *SKP2B*, an F-box protein that regulates lateral root formation and it is expressed in lateral root founder cells and lateral root primordia (*6*). Histochemical detection of GUS activity was performed using 6-d-old plants directly germinated on agar-plates containing 0.5x MS, 1% bacto-agar (Sigma) in axenic conditions or with the bacterial isolates. Roots were treated with 90% acetone on ice for 1h and then with 5-bromo-4-chloro-3-indolyl-b-D-glucuronide (X-Gluc) buffer solution (1 mM X-Gluc, 100 mM $NaPO_4$ (pH 7), 2 mM $K_3[Fe(CN)]_6$, 2 mM $K_4[Fe(CN)]_6$, 1 mM EDTA) for 6 h at 37°C. Roots were finally fixed in 20% methanol and 4% hydrochloric acid for 15 min. Then, roots were cleared using 60% ethanol in 7% NaOH for 15 min followed by successive soaks in 40% ethanol, 20% ethanol, 10% ethanol for 5 min. Samples were stored in 50% glycerol until use. For

analysis, roots were placed on microscope slides and covered with a coverslip. Lateral root primordia in the entire root were quantified using a Leica 2 DM5000B fluorescence microscope at 40X magnification.

h. Visualization of lateral root primordia

For the visualization of the bacteria effect on lateral root primordia we used the reporter line *pLBD16:GFP* (*7*). This line expresses the green fluorescent protein (GFP) under the promoter of the lateral root regulator *LATERAL ORGAN BOUNDARIES DOMAIN16* (*LBD16*), that is expressed in lateral root primordia. 6-d-old *pLBD16:GFP* seedlings, directly germinated with individual bacteria strains or with 10 mM $MgCl_2$ (No Bacteria (NB) control), were placed on microscope slide with water, covered with a coverslip, and GFP expression was visualized using a Leica SP8 confocal microscope, 40X objective (NA = 0.8) with an excitation at 488 nm and an emission band-path of 500–550 nm. Longitudinal sections of roots were imaged from the root tip.

i. Pre-branch sites quantification

For the visualization of *DR5:Luciferase* activity, plates containing 6-d-old *DR5: Luciferase* seedlings exposed or not to the different bacterial treatments were sprayed with 5 mM Beetle Luciferin (Promega) and then imaged for 4 min using a Lumazone CA Automated Chemiluminescence System (Roper Bioscience). These images were used to quantify the total primary root length and the number of pre-branch sites, using Image J (*33*).

j. Quantification of changes in auxin signaling.

To determine whether the bacteria isolates change the auxin signaling in the root we used the auxin sensor *DII:VENUS* (*32*). This construct expressed the VENUS fast maturing yellow fluorescent protein fused to the auxin-interaction domain (DII) of IAA28 under the

constitutive *35S* promoter (*32*). Thus, in the presence of low levels of auxin the VENUS signal is detected. Six-day-old *DII:VENUS* seedlings, directly germinated with the individual bacteria strains or with 10 mM $MgCl_2$ (No Bacteria (NB) control), or 100 nM IAA as a positive control, were placed on microscope slide with water, covered with a coverslip, and the VENUS expression was visualized using a Leica SP8 confocal microscope, 40x objective (NA = 0.8) with an excitation at 514 nm and an emission band-path of 519–569 nm. Root tissues were visualized in the confocal microscope using propidium iodide staining with an excitation at 514 nm and an emission band-path of 608–694 nm. To quantify VENUS fluorescence, serial optical sections of the root, including the root tip, were obtained. Image z-stacks were taken about the midline of roots to include all nuclei required for quantification. Then, 2D planes were generated from the 3D stacks using the SurfaceProject plug-in for the Fiji image analysis package (*37*). The VENUS fluorescence intensities were quantified in all 2D planes using the ROI tool of Image J (*33*).

k. NPA and L-Kynurenine as bacterial carbon sources.

To test if the bacterial isolates can use N-1-naphthylphthalamic acid (NPA, an inhibitor of auxin polar transport, (Sigma)) and L-kynurenine (L-Kyn, an inhibitor of auxin biosynthesis, (Sigma)) (*38*, *39*) as carbon sources, we grew the bacterial isolates in M9 minimal salts medium (Sigma) supplemented with 2 mM $MgS0_4$, 0.1 mM $CaCl_2$, 10 µM $FeSO_4$, and either 50 µM NPA or 15 µM L-Kyn, or 15 mM succinate (Sigma) as carbon sources. All cultures were grown for two days at 28°C with agitation (250 r.p.m). This time was sufficient to reach the stationary phase of all culture used. In all cases, the bacterial growth was monitored by measure the OD at 600nm using a FLUOstar® Omega (BMG LABTECH).

l. Computational and statistical analysis.

To analyze the effect of bacteria on *Selaginella* branching, we first reduced biases caused by skewness and outliers in the data by transforming the measured bifurcation events by applying the $\log_{10}$ followed by z-score standardization across the measurements of all tested strains.

We determined differences in the magnitude of bifurcation events against the no bacteria (NB) treatment via the fitting of a linear model with the formula:

Standardized number of bifurcation events ~ Strain

Statistical significance with respect to the NB treatment were performed via the Dunnet test implemented in the glht function from the multcomp v.1.4-19 R package (*27*).

We compared the effect of bacterial isolates, harboring or lacking auxin biosynthetic and degradation operons, on *Selaginella* branching by determining the distribution of estimates (with respect to the no bacteria (NB) treatment) of both type of isolates (Operon presence vs absence). Differences between the distributions of effects across types of isolates were determined via the Kolmogorov-Smirnov test coded in the function ks.test from the stats package of R (*27*).

The ability of each bacterial strain to grow on minimal M9 medium supplemented with succinate was quantified from the individual bacterial growth curves. Briefly, for each of the three replicate per strain, we calculated the slope of the exponential phase of the growth curves. Estimated negative slopes were assigned a slope value of zero denoting no growth. For each evaluated strain, we averaged the slopes across three replicates and represented them via a heatmap.

For the analysis of bacterial auxin production and degradation under in vitro conditions, we compared the IAA content, measured as absorbance, for each bacterial strain against values obtained using *Escherichia coli* with a linear model approach. Statistical significance with respect to the *Escherichia coli* treatment was performed via the Dunnet test implemented in the glht function from the multcomp v.1.4-19, R package (*27*).

Primary root elongation was compared between the plant genotypes used by taking solely measurements obtained from the no bacteria (NB) treatment and fitting a linear model with the following design:

Primary root elongation ~ Genotype

Statistical significance across plants genotypes was determined via the emmeans function from the emmeans v.1.7.3 R package and represented through the compact letter display via the cld function from the multcomp v.1.4-19, R package (*27*).

To analyze the lateral root density across the plant genotypes exposed to the different bacterial treatments, we only used plants that produced 4 or more lateral roots across the genotypes used. This helped discard spurious bacterial effect on lateral roots. We reduced the skewness within the measurements by transforming the lateral root density values applying the $\log_{10}$ followed by intra-genotype z-score standardization. Next, within each genotype, we evaluated the ability of each strain to modify the lateral root density with respect to the no bacteria (NB) treatment via the fitting of a linear model with the following design:

Standardized lateral root density ~ Strain

Statistical significance with respect to the NB treatment was performed via the Dunnet test implemented in the glht function from the multcomp v.1.4-19, R package (*27*). Estimated *p*-values were false discovery rate (FDR) corrected.

For the ionomic analysis, we constructed matrices with the ion concentrations across samples. We then transformed the values within matrices by applying the $\log_{10}$ to the raw data followed by intra-ion z-score standardization to compare differences across the dissimilar magnitudes of the ions.

For the ionomic matrix in the MS medium dilution experiment, we visualize the treatment-specific ionomic profiles via principal component analysis (PCA) using the prcomp function in R (*27*). Additionally, these data were visualized in a heatmap by averaging per treatment the estimated concentration of all individual ions across replicates in the dataset. The heatmap was subjected to hierarchical clustering using Euclidean distance and ward.D clustering method implemented in the rcorr function from the Hmisc v4.6.0, R package (*27*) across both, rows (ions) and columns (treatments).

For the correlation between lateral root density and endodermal suberization, we retrieved the average strain-induced changes in suberization previously published (Figure 2*A* from Salas-Gónzalez *et al* (*4*)). In parallel, we calculated the average lateral root density measured for each of the plants inoculated with individual bacterial strains showed in Fig. 1*D*. We correlated both parameters by plotting in a scatterplot the average estimated suberization (sum of no expression and discrete zones; Figure 2*A* from Salas-González *et al* (*4*)) against the average lateral root density measured for the 378 bacterial strains analyzed in both screenings. The Pearson correlation coefficient between both

parameters was computed and displayed via the stat_cor function from the ggpubr package v.0.4.0.

To determine the effect of the bacterial isolate RMF27 on lateral root development in the line *pCASP1::CDEF1* (*5*), we compared the observed lateral root density across treatments (wild-type plants and the line *pCASP1::CDEF1* exposed or not to RMF27) by fitting a linear model followed by group contrasting through the emmeans function from the emmeans v.1.7.3, R package. Difference between groups was represented through the compact letter display via the cld function from the multcomp v.1.4-19, R package (*27*).

For the analysis of the primordia counted in wild-type and mutant plants using the microscope, we summed the total number of primordia (independently of the primordia stage) counted for each plant analyzed. Then, for each individual plant, we calculated the relative abundance of primordia at each stage by dividing the number of primordia for that stage by the total number of primordia detected.

In order to compare the effect of each bacterial strain on the total number of primordia, we fitted a Poisson-family generalized linear model (GLM) within each genotype with the following formula:

$$\text{Number of primordia} \sim \text{Strain}$$

Statistical significance with respect to the no bacteria treatment (NB) within each genotype was determined via the Dunnet test implemented in the glht function from the multcomp v.1.4-19, R package (*27*). The average relative abundance of each primordial stage across the bacterial strains analyzed was represented as a stacked bar graph plotted via ggplot2 v3.3.5.

For the primordia counted using the reporter line *pSKP2B::GUS* (*6*), we summed the total number of primordia (independently of the primordia stage) analyzed for each plant. Then for each plant we computed the relative abundance of the primordia at each stage by dividing the number of primordia counted for that stage by the total number of primordia. The average relative abundance of each primordia stage across the bacterial strains analyzed was represented as a stacked bar graph plotted via ggplot2 v3.3.5.

To correlate the primordia counted in wild-type plants using the microscope and the primordia counted in the *pSKP2B::GUS* line (*6*), we took as an unit the primordia stage detected per individual bacterial strain in both cases. We visualized this correlation via a scatterplot over which we overlaid the Pearson correlation coefficient between both features computed and displayed via the stat_cor function from the ggpubr package v.0.4.0.

Finally, for the primordia counted in wild-type plants using the microscope, we estimated a correlation coefficient for the total number of primordia counted across all bacterial strains and the average primordia observed at each stage. We pooled the data across all bacterial strains to perform this correlation. Significance at each stage was computed and displayed via the stat_cor function from the ggpubr package v.0.4.0.

To determine differences in pre-branch sites densities across bacterial treatments, we compared the results of individual bacterial treatments against the no bacteria (NB) treatment by fitting of a Kruskal-Wallis test implemented in the Kruskal.test R function with the following design:

Pre-branch sites density ~ Strain

The *p*-value between each strain and NB treatments was adjusted for multiple testing correction using the Bonferroni method. We visualized the results via a boxplot using the ggplot R package (*27*).

To analyze the fluorescence in the auxin sensor *DII:VENUS*, we quantified the median fluorescence across all pixels measured from the images. We evaluated the ability of each bacterial strain to change the fluorescence levels in the auxin sensor line relative to the no bacteria (NB) treatment via the fitting of a linear model with the following design:

Fluorescence ~ Strain

Statistical significance with respect to the NB treatment was performed via the Dunnet test implemented in the glht function from the multcomp v.1.4-19 R package (*27*). Estimated *p*-values were false discovery rate (FDR) corrected.

To determine the effect of L-Kyn and NPA on lateral root development, we compared the lateral root density in plants treated with the bacterial strain co-inoculated along with L-Kyn or NPA with respect to the lateral root density in plants inoculated with the bacterial strains alone (No molecule). Statistical significance between the L-Kyn and NPA treatments with respect to the No molecule one was assessed via the Dunnet test implemented in the glht function from the multcomp v.1.4-19, R package (*27*) taking as input the fitting of a linear model with the formula:

Lateral root density ~ Molecule

For visualization purposes, we calculated for each bacterial strain treatment, the ratio between the average lateral root density observed in NPA and L-Kyn treated plants against the average lateral root density in plants inoculated with this bacterial strain alone (No molecule).

The ability of each bacterial strain to grow in a minimal medium M9 supplemented with Succinate, L-Kyn, or NPA was quantified from individual bacterial growth curves. Briefly, for each of the three replicate per bacterial strain used, we calculated the slope of the exponential phase of the individual growth curves. Estimated negative slopes were assigned a value of zero denoting no growth. The average of the slopes across individual bacterial strains was represented via a heatmap.

3. Experiments to define the molecular mechanism coordinating plant microbiota and root branching

a. Plant growth conditions

To define the mechanism that plant use to coordinate with the plant microbiota during lateral root development, we analyzed the effect of the selected individual bacterial isolates, hormonal treatments, and hormonal inhibitors on lateral root development of *Arabidopsis* wild-type plants, hormonal mutants, reporter lines, and also on the basal plant *Selaginella*.

Sterilized Col-0, lateral root mutants (*arf7 arf19*, *nph4-1*, *lbd16-1*, and *gnom184*), ethylene mutants (*ein3 eil1, ein2, etr1, ctr1*, *eto3, mtk1*), and defense mutant *fls2* seeds were first germinated on agar plates containing 0.5x MS (Sigma) medium solidified with 1% bacto-agar (Sigma) for seven days. Then, approximately ten seedlings with similar development were transferred to new 0.5x MS agar plates inoculated with the individual isolates alone or plates supplemented with 5 µM NPA or 1.5 µM L-Kyn, or 10 µM of the ethylene precursor 1-Aminocyclopropane-1-carboxylate (ACC), or 2 µM of the ethylene biosynthesis inhibitor Aminoethoxyvinylglycine (AVG) (*40*) or plates inoculated with bacterial strains together with one or two of the chemicals described. As a control we also transferred ten seedlings to a plate inoculated with only 10 mM $MgCl_2$ with no bacteria

present (no bacteria (NB) control). Plates were manually randomized and placed in a growth chamber and grown in a vertical position under a 16-h light/8-h dark regime at 21°C day/19°C night for another 7 d. We used 2 replicates of each bacterial treatment and this experiment was repeated at least twice.

Sterilized seeds of the ethylene reporter construct *EBS:GUS* that expresses the *β-Glucuronidase* (*GUS*) reporter gene under the control of a synthetic *EIN3*-responsive promoter (*39*) were direct germinated on agar-plate containing 0.5x MS (Sigma), 1% bacto-agar (Sigma) inoculated or not with the selected bacterial strains, or supplemented with 10 µM ACC as control, for six days.

ACC was extracted and quantified according to the method of Bulens et al (2011) (*41*). Briefly, root tissue was collected from several plants and pooled to have sufficient biomass per replicate and ten replicates were analyzed per treatment. The tissue was immediately ground in liquid nitrogen. ACC was extracted from 200 mg frozen powder using 80% cold ethanol for 30 min, centrifuged and the supernatant was evaporated under vacuum. The pellet was dissolved in 440 µL of water and analyzed for ACC content according to Bulens et al. (2011) (*41*), by converting ACC into ethylene gas, which was subsequently quantified by gas chromatography (Shimadzu GC2014).

Selaginella plants were grown axenically from explants in closed magenta jars containing 0.5x MS (Sigma) medium solidified with 0.4% bacto-agar (Sigma) under a 16-h light/8-h dark regime at 21°C day/19°C night for two months, sufficient time to generate fully developed *Selaginella* plants. New *Selaginella* explants were prepared from the magenta cultures using scalpel and forceps under sterile conditions and approximately 12 explants of similar sizes, lacking roots were then transferred to agar plates with 0.5x MS, 0.8%

bacto-agar (Sigma) inoculated with the bacterial strain RMF27 or to plates supplemented with 10 μM ACC, or 2 μM AVG or combinations of them. As a control we also transferred *Selaginella* to no bacteria (NB) treatments, plates inoculated with 100 μL of 10 mM $MgCl_2$ and supplemented or not with ACC or AVG. Plates were randomized, placed in a growth chamber, and grown in a vertical position under a 16-h light/8-h dark regime at 21°C day/19°C night for 14 d.

b. RNA extraction

In all cases, RNA was extracted from plant roots following Logemann et al. (*42*). Briefly, wild-type and mutant seeds were germinated on agar plates under axenic conditions for seven days and then transferred to plates inoculated or not with the bacterial isolates and let them grow for another seven days. Approximately 10 roots from 14-d-old seedlings were harvested from each sample and flash frozen using liquid nitrogen. Frozen tissue was pulverized in a TissueLyzer II (Qiagen), using 2 cycles of 30 seconds, frequency 30 s^{-1}. We added to each sample 400 μL of Z6-buffer (8 M guanidine HCl, 20 mM MES, 20 mM EDTA at pH 7.0). Then, samples were treated with 400 μL phenol:chloroform:isoamylalcohol; 25:24:1 (Sigma), vortexed, and centrifuged (20,000 g, 10 min) to separate the phases. The aqueous phase, containing the RNA, was transferred to a clean 1.5-mL Eppendorf tube and the RNA was precipitated by adding 0.05 volumes of 1 N acetic acid and 0.7 volumes 96% ethanol and incubating at −20°C, overnight. Samples were centrifugated at 20,000 g, 10 min, 4°C, and the pellet was washed sequentially with 200 μL sodium-acetate (pH 5.2) and 70% ethanol. The RNA was dried for ten minutes, resuspended in 30 μL of ultrapure water, and stored at −80°C until use. We used three independent biological replicates per treatment and we repeated this experiment twice.

c. Real-time PCR analysis

For RT-PCR analysis, all RNA samples were first DNase treated with DNAse I (Thermo Scientific) and then tested for the absence of genomic DNA by PCR, with primers spanning an exon junction. cDNA synthesis was performed using RevertAid First Strand cDNA Synthesis Kit (Thermo Scientific) according to the manufacture's recommendations. Primers for the gene targets analyzed were designed using the National Center for Biotechnology Information Primer-BLAST (Dataset S7). Gene expression was determined using SensiMix™ SYBR® Hi-ROX Kit (Bioline) and the relative expression across treatments and genotypes were determined using the housekeeping gene *elongation factor-1α* (*EF-1α*) (At1g07940) for normalization and a comparative quantification method. We used six biological replicates per treatment.

d. Plant RNA sequencing

Isolated RNA was treated with Turbo DNA-free (Applied Biosystems), according to the manufacturer's instructions to remove contaminating DNA. RNA libraries were then prepared using 1 µg RNA following Finkel et al (*30*) with modifications. Briefly, mRNA was purified from total RNA with Sera-mag oligo(dT) magnetic beads (GE Healthcare Life Sciences) and fragmented at 94 °C for 6 min. Fragmented mRNA was used for first-strand cDNA synthesis using reverse transcriptase and random hexamers, then the second-strand cDNA was synthesized using DNA polymerase I and the contaminating RNA removed using RNaseH. Double-stranded cDNA was end-repaired using T4 DNA polymerase, T4 polynucleotide kinase, and Klenow polymerase. The adenylation of the DNA fragments were performed using Klenow exo-polymerase to allow the ligation of KAPA Dual-Indexed adapters (KK8722).

Library yield was measured by Qubit dsDNA HS (Invitrogen; Q32851) and library size was assessed using the High Sensitivity D1000 ScreenTape (Agilent; 5067- 5584) on the Agilent 4200 TapeStation System (Agilent; G2991A). Equimolar quantities of individual barcoded RNA libraries were pooled (3 pools) in a randomized manner, and shipped in dried ice to Beijing Genomics Institute, Shenzhen, China. Each library pool was sequenced on three lanes (for a total of nine lanes) on a MGI Tech MGISEQ-2000 sequencing platform to generate a minimum of 10 million paired-end, 100 bp reads per sample.

e. Bacterial colonization analysis

To re-isolate and quantify bacteria strains across the different treatments we used the colony-forming unit (c.f.u) method. At least four roots were collected separately from 14-d-old plants. Roots were weighted in previous weighted sterile 2-mL Eppendorf tubes containing 3 glass beads. Samples were then washed three times with 10 mM $MgCl_2$ and 200 µL of 10 mM $MgCl_2$ was added to each sample. All samples were homogenized in a TissueLyser II (Qiagen) using 2 cycles of 30 seconds, frequency 30 s^{-1}. Finally, 20 µL of each sample was serially diluted in 96-wells plates in 180 µL of 10 mM $MgCl_2$. To re-isolate and quantify bacterial strains used, 5 µL of every single dilution was plated on fresh prepared square 12 x 12 plates (Greiner Bio-One) containing LB medium solidified with 1% bacto-agar (Sigma) and grown at 28°C overnight. The number of c.f.u were determined from the plates using a Leica MZ 10F stereo microscope. The final c.f.u/mL values in each sample were determined considering the dilution factors and normalized across treatments by the sample weight. We used at least three biological replicates per treatment.

f. Histochemical detection of GUS activity

To monitor the levels of ethylene in the root we used the ethylene reporter construct *EBS:GUS*. In this line, the *β-Glucuronidase* (*GUS*) reporter gene is expressed under the control of a synthetic *EIN3*-responsive promoter (*43*). Histochemical detection of GUS activity was performed using 6-d-old plants direct germinated on agar-plates containing 0.5x MS, 1% bacto-agar (Sigma) in axenic conditions or with the bacterial isolates. Roots were treated with 90% acetone on ice for 1h and then with 5-bromo-4-chloro-3-indolyl-b-D-glucuronide (X-Gluc) buffer solution (1 mM X-Gluc, 100 mM $NaPO_4$ (pH 7), 2 mM $K_3[Fe(CN)]_6$, 2 mM $K_4[Fe(CN)]_6$, 1 mM EDTA,) for 6 h at 37°C. Roots were finally fixed in 20% methanol and 4% hydrochloric acid for 15 min. Then, roots were cleared using (60% ethanol in 7% NaOH) for 15 min followed by successive soaks in 40% ethanol, 20% ethanol, 10% ethanol for 5 min. Samples were stored in 50% glycerol until use. For analysis, roots were placed on microscope slide and covered with a coverslip. Images of the GUS expression pattern in the root tip in response to bacterial isolates or to axenic conditions were taken in a fluorescence microscope Leica 2 DM5000B, 5x magnification. We included plants treated with 10 µM ACC as a control. These images were used to quantify, using Image J (*33*), the distance from the root tip to the continuous detection of GUS activity in the root vasculature as a proxy for ethylene accumulation. This distance is inversely proportional to the amount of ethylene in the root.

g. ACC and AVG as bacterial carbon sources.

To determine whether the bacterial isolates can use the ethylene precursor 1-Aminocyclopropane-1-carboxylate (ACC) and the ethylene biosynthesis inhibitor Aminoethoxyvinylglycine (AVG) (*40*) as carbon sources, we grew the bacterial isolates in M9 minimal salts medium (Sigma) supplemented with 2 mM $MgS0_4$, 0.1 mM $CaCl_2$, 10 µM $FeSO_4$, and either 100 µM ACC or 20 µM AVG, or 15 mM succinate (Sigma) as carbon sources. All cultures were grown for two days at 28°C with agitation (250 r.p.m). This time

was sufficient to reach the stationary phase of all culture used. In all cases, the bacterial growth was monitored by measure the OD at 600nm using a FLUOstar® Omega (BMG LABTECH).

h. Split root experiments.

To determine the effect of the volatile compounds produced by the bacteria isolates on lateral root formation, we performed split root assays. For this experiment, plants were germinated under axenic conditions on square plates (Greiner Bio-One) containing 0.5x MS, 1% bacto-agar (Sigma) for seven days and then transferred to split plates (Sarstedt) for another 7 d. The split plates have two compartments physically separated by a plastic barrier that prevents the media contact but not the gas exchange between compartments. For each bacterium we designed four treatments: (plant + no bacteria) *vs* (no plant + no bacteria); (plant + bacteria) *vs* (no plant + bacteria); (plant + bacteria) *vs* (no plant + no bacteria); (plant + no bacteria) *vs* (no plant + bacteria). We determined the primary root length and the number of secondary roots across all conditions from images taken using a linear robot camera.

i. Branching parameters determination

We determined the primary root length and the number of lateral roots in wild-type and mutant plants from images taken using a linear robot camera. Primary root length and the number of lateral roots were measured using Image J (*33*) and SmartRoot software (*20*). The density of lateral roots was calculated dividing the number of lateral roots by the length of the primary root.

The number of bifurcation events in *Selaginella* in response to different treatments was determined directly from the plates using a dissecting microscope. We considered that a root was bifurcated when a clear Y was formed at the extremity of the roots.

j. Computational and statistical analysis

For the RT-PCR analysis, we defined a set of auxin and plant defense markers from the literature. In the case of auxin, we selected genes that were up-regulated in at least six out of seven datasets analyzed (*44*, *8–12*) and exhibited a gene ontology related with auxin metabolism. For the defense markers we selected the genes from previous publications (*16*, *17*). For each of the genes measured, we standardized their relative expression (scale function from the stats R package (*27*) across all treatments. This allows us to reduce the differences in magnitude across genes and better visualize differences across bacterial treatments in a similar scale.

For the analysis of the RNA-Seq data, reads were first subjected to quality control and filtering via fastp v0.20.1 (*45*). The resulting high-quality reads were pseudo-aligned to the *Arabidopsis* genome deposited in the Ensembl Plants version 52, using salmon v1.6.0 (*46*). Gene-level summarization from the salmon quantification was performed with the tximport function from the tximport v.1.22.0 R package (*27*).

We used DESeq2 v.1.34.0, R package (*27*) to identify a set of differentially expressed genes across treatments. To do so, we grouped both of the design variables (bacterial treatment and genotype) into a new grouping variable (e.g. Col-0_RMF27 or *gnom184*_L469 or *arf7 arf19*_NB) and fitted the following generalized linear model (GLM):

$$\text{Gene Abundance} \sim \text{group}$$

Next, we set up the following contrasts to identify sets of differentially expressed genes (DEGs) with specific types of effects:

1) Bacterial effect in Col-0: We compared each of the bacterial strains inoculated in Col-0 against Col-0 grown axenically. (e.g. Col-0_L180 *vs* Col-0_NB, Col-0_RMF27 *vs* Col-0_NB, etc).

2) Bacterial effect within each mutant: We compared each of the bacterial strains inoculated in a given mutant against the axenic treatment of the corresponding mutant. (e.g. *gnom184*_L339 *vs gnom184*_NB, *nph4-1*_L344 *vs nph4-1*_NB).

3) Gene expression association with lateral root density: We applied a variance stabilizing transformation to the raw count gene matrix followed by the standardization of the expression of each gene along the samples in the dataset. For each group (Genotype_Strain, e.g. Col-0_RM27 or *gnom184*_L339), we calculated the average expression of each gene. In parallel, for each of the bacterial (including the NB control) treatments in Col-0, we calculated the average lateral root density observed in each given treatment. Finally, using the function cor.test from the stats R package (*27*), we correlated for all measured gene expression its correlation with the lateral root density across all bacterial treatments within Col-0.

We devised a combinatorial scheme based on the three contrasts described above to subclassify DEGs into groups with specific functional interpretations. Briefly, for each of all the 16 bacterial treatments across mutants (*lbd16-1*_L180, *gnom184*_L196, *gnom184*_L339, *lbd16-1*_L343, *arf7 arf19*_L344, *nph4-1*_L344, *lbd16-1*_L359, *nph4-1*_L359, *lbd16-1*_L384, *lbd16-1*_L412, *gnom184*_L469, *lbd16-1*_L76, *nph4-1*_RCL115,

*lbd16-1*_RMF217, *gnom184*_RMF27, *lbd16-1*_RMF8) we extracted all DEGs across their respective three contrasts. For example, for the treatment *lbd16-1*_L180:

1) We extracted all DEGs in the contrast Col-0_L180 *vs* Col-0_NB. Genes were labelled as -, if they were statistically significantly more expressed in Col-0_NB than in Col-0_L180. Genes were labelled as +, if they were statistically significantly more expressed in Col-0_L180 than in Col-0_NB.

2) We extracted all DEGs in the contrast *lbd16-1*_L180 *vs* *lbd16-1*_NB. Genes were labelled as -, if they were statistically significantly more expressed in *lbd16-1*_NB than in *lbd16-1*_L180. Genes were labelled as +, if they were statistically significantly more expressed in *lbd16-1*_L180 than in *lbd16-1*_NB.

3) We extracted all genes that exhibit a significant positive correlation with the lateral root density across Col-0 and labelled them as +. Then, all genes with a significant negative correlation with the lateral root across Col-0 were labelled as -.

Once we identified and labelled (-,+) all DEGs in the three categories above described, we computed the combinatorial intersections among the three categories. This allows us to label each DEG in the treatment (In this exemplary case *lbd16-1*_L180) using a three digit code that could take three values in each position: [-0+][-0+][-0+]. For example, a gene with the code [+++] would represent a gene that was up-regulated (with respect to the NB treatments) in response to a bacterium in Col-0 and in a given mutant, and that exhibited a significant positive correlation with the lateral root density. A gene with the code [---] would represent a gene that was down-regulated (with respect to the NB treatments) in response to a bacterium in Col-0 and a given mutant, and that exhibited a

significant negative correlation with the lateral root density. The results of this classification scheme are depicted in *SI Appendix,* Fig. S7C as alluvial plots computed using the ggaluvial v0.12.3 R package (*27*).

To narrow down the list of candidate genes, we performed hierarchical clustering over the union of genes (across all the 16 treatments mentioned above) that exhibited either a pattern ++[0+] or –[-0] (*SI Appendix,* Fig. S7D). We established the clusters within this heatmap via the dendrogram partitioning algorithm implemented in the function cutree from the stats R package (*27*).

Gene ontology analysis was performed over each of the delimited clusters employing the function compareCluster function from the clusterProfiler v.4.2.2, R package (*27*). Visualization of the gene ontology enrichment was performed using the cnetplot function from the clusterProfiler v.4.2.2, R package (*27*).

The heatmap presented in Fig. 4*D* was built using a subset of genes from *SI Appendix,* Fig. S7*D*; genes exhibiting significant correlation with the lateral root density phenotype. This heatmap was subjected to hierarchical clustering as described before.

The core gene sets used in this work was identified from the literature. In the case of ethylene (*4*), flg22 (*14*), and Cytokinin (*13*) we used gene sets previously published. For auxin, we defined a set of robust auxin markers by selecting genes that were found significantly induced in at least four out of six datasets used (*8–12*).

To examine the enrichment of the hormone and flg22 core gene sets across the filtered genes that respond to the bacteria presence and correlated with lateral root density (Fig.

4*A*), we applied a hypergeometric test. The *p*-values of each test were adjusted using the FDR method. We considered an enrichment as significant if it had an adjusted *p*-value < 0.05. We repeated the same analysis solely using genes identified as transcription factors.

To perform the sequential (log2fold change) enrichment of hormonal and flg22 core genes, we took within Col-0 the average log2 fold change for all bacterial treatments with respect to the no bacteria (NB) treatment. Then, we established sets of genes based on sequential cutoffs of average log2fold change. Enrichment of the sets were tested using the hypergeometric test described above.

To identify the tissue-specific expression profile of the 28 ethylene-related genes induced in response to the bacteria colonization with high correlation with lateral root density, we downloaded the spatial expression profile of each gene from the Klepikova atlas (*15*) using the bio-analytic resource of plant biology platform (BAR) (*47*). Then, we constructed a spatial expression matrix of these 28 genes and computed pairwise Pearson correlation between all pairs of genes. Finally, we applied hierarchical clustering to this correlation matrix and the results were visualized in a heatmap.

We compared the effect of each genotype/molecule/split root treatment over each bacterial strain ability to influence lateral root density via the fitting of a linear model within each bacterial strain treatment with the following formula:

Lateral root density ~ Genotype/Molecule/Split root treatment

Statistical significance across treatments was determined via the emmeans function from the emmeans v.1.7.3, R package (*27*) and represented through the compact letter display via the cld function from the multcomp v.1.4-19, R package (*27*).

For the analysis of the bacterial colonization data related to the genotypes Col-0, *ein3 eil1*, *ein2,* and *etr1*, we fitted a linear model taking as dependent variable the logarithm-transformed base 10 c.f.u. values. Statistical significance across plants genotypes was determined via the emmeans function from the emmeans v.1.7.3, R package (*27*) and represented through the compact letter display via the cld function from the multcomp v.1.4-19, R package (*27*).

For the analysis of the lateral root density and bacterial colonization data obtained for mutants *mtk1* and *fls2*, we performed intra-strain comparison between genotypes utilizing the t.test function from the stats R package (*27*).

In addition to comparing the genotype effect (e.g. *ein3 eil1, ein2,* and *etr1*) within each bacterial strain, we compared the overall genotype effect across all bacterial strains via plotting the distribution of standardized lateral root densities per genotype. To do so, we standardized, using the scale function from the stats R package (*27*), the lateral root densities measured within all samples inoculated with a given strain (e.g. RMF27). This standardization permitted us to remove magnitude differences across bacterial strains and to focalize the genotype effect. We visualized the density distributions utilizing the ggridges v.0.5.3, R package (*27*).

We repeated the same density comparison scheme for the experiment evaluating the effect of ACC and AVG across bacterial strains within distinct genotypes. For each genotype and within each strain in that genotype, we standardized, using the scale function from the stats R package (*27*), the lateral root densities measured within all samples inoculated with that given bacterial strain. We visualized the density distributions utilizing the ggridges v.0.5.3, R package (*27*).

4. Experiments with synthetic and natural communities

a. Natural microbiota isolation from soil

Soil natural microbial populations were isolated from a natural soil from Sutton-Bonington Campus (University of Nottingham, UK; +52° 49′ 59.75″N, −1° 14′ 56.62″W). Approximately 800 mL of soil was mix vigorously with 1 L of autoclaved RO water to bring microbes into suspension. The resulting solution was allowed to settle for 20 minutes to allow large soil particles to precipitate. Then, the supernatants were carefully filtered using a sterile funnel lined with sterile miracloth in a laminar flow cabinet. The filtrated solutions containing the soil microbes were incubated for another 20 min. The solution was then spin in a centrifuge (Eppendorf 5810R) 3220 g at room temperature for 30 min. The supernatants were discarded and the pellets were resuspended in 100 mL of MES pH 6. Soil solutions were centrifuged again at 3220 g at room temperature for 30 min. The supernatants were discarded and the pellets resuspended in 100 mL MES pH 6.0 with vigorous shaking and/or vortexing. This centrifugation and resuspension step was repeated once. The soil solution was centrifuged at 3220 g at room temperature for 30 min and the pellets were resuspended in 20 mL MES pH 6.0 in a sterile 50-mL falcon tube. This final soil solution containing the soil natural microbial community were incubated on ice until use. The natural community inoculum (100 µL) was spread using a L-shaped cell spreader (Fisher scientific) on the surface of 12 X 12 cm square agar plates prior to transferring seedlings.

b. Bacterial synthetic community preparation

The bacterial synthetic community was designed using 16 bacterial strains that cover the diversity of the bacterial effect on lateral root development. These bacterial isolates belong to two collections of bacterial strains: one isolated from roots and one isolated from leaves.

The root collection was prepared using only roots of Brassicaceae (mostly *Arabidopsis*) grown in two natural soils from USA (*18*). For the leaf collection, bacterial strains were isolated from leaves of healthy *Arabidopsis* plants collected from six locations around Tübingen, Germany, or Zurich, Switzerland (*19*). All bacterial cultures were grown from single colonies. To isolate single colonies from the bacterial isolates, -80°C glycerol stocks were grown on LB in the case of root collection and R2A for the leaf collection plates incubated at 28°C. The R2A medium composition is Casein acid hydrolysate 0.5 g/L, Yeast extract 0.5 g/L, Protease peptone 0.5 g/L, Dextrose 0.5 g/L, Starch 0.5 g/L, Dipotassium phosphate 0.3 g/L, Magnesium sulfate 0.024 g/L, Sodium pyruvate 0.3 g/L, agar 15g/L, pH 7.2 supplemented with 0.5% Methanol. Once isolated, a single colony was then inoculated in sterile 15-mL falcon tubes with approximately 4 mL of LB medium (root collection) or R2A medium supplemented with 0.5% Methanol (leaf collection) and grown in an incubator at 28°C with agitation at 250 r.p.m. To remove media used and cell debris, all cultures were centrifuged in a benchtop centrifuge (Eppendorf 5810R), 3220 g at 4°C, and washed three times with 10 mM $MgCl_2$. Finally, clean bacterial cells were resuspended in 1mL of 10 mM $MgCl_2$, and the OD_{600nm} was measured. To equalize bacterial cultures, we assumed that 1 OD_{600nm} unit is equal to 10^9 c.f.u/mL, and individual bacteria were mixed at a final concentration of 10^5 c.f.u/mL in the synthetic community. The synthetic community inoculum was adjusted to an OD_{600nm} = 0.2 and 100 µL of the synthetic community was spread using a L-shaped cell spreader (Fisher scientific) on the surface of 12 x 12cm square agar plates (Greiner Bio-One) prior to transferring seedlings.

c. In vitro plant growth conditions

To determine the role of the plant microbiota in controlling the formation of lateral roots we analyzed the lateral roots density in wild-type Col-0, lateral root mutants *arf7 arf19 and gnom184*, and the ethylene mutants *ein3eil1,* in response to a natural and a synthetic

community of microbes under full nutrient conditions or abiotic stresses. Sterilized Col-0 and mutant seeds were germinated for seven days on agar plates containing 0.5x MS medium solidified with 1% bacto-agar (Sigma). Then, approximately ten seedlings were transferred to agar plates inoculated with the 16-members synthetic community or a natural soil microbial community in full medium or under two different nutritional stresses. To impose the stresses to the plants, the composition of the 0.5x MS medium (macro-elements: NH_4NO_3 (825 mg/L), $CaCl_2$ (166.1 mg/L), $MgSO_4$ (90.345 mg/L), KNO_3 (950 mg/L), KH_2PO_4 (85 mg/L), micro-elements: H_3BO_3 (3.1 mg/L), $CoCl_2 \cdot 6H_2O$ (0.0125 mg/L), $CuSO_4 \cdot 5H_2O$ (0.0125 mg/L), $Na_2EDTA \cdot 2H_2O$ (18.65 mg/L), $FeSO_4 \cdot 7H_2O$ (13.9 mg/L), $MnSO_4 \cdot H_2O$ (8.45 mg/L), $Na_2MoO_4 \cdot 2H_2O$ (0.1065 mg/L), KI (0.415 mg/L), $ZnSO_4 \cdot 7H_2O$ (4.3 mg/L), vitamins: myo-Inositol (50 mg/L), Nicotinic acid (free acid) (0.25 mg/L), Pyridoxine-HCl (0.25 mg/L), Thiamine hydrochloride (0.05 mg/L), amino acids: Glycine (1 mg/L, pH 5.6 - 5.7)) in the agar plates was amended. These amendments included Fe-free medium (without FeSO4·7H2O), and 0.5x MS medium supplemented with 100 mM NaCl. In parallel, 10 seedlings were also transferred to agar plates containing the no bacteria (NB) control (only 10 mM $MgCl_2$). Plates were manually randomized and placed in a growth chamber and grown in a vertical position under a 16-h light/8-h dark regime at 21°C day/19°C night for another 7 d.

d. Root branching and shoot parameters quantification

We determined the primary root length and the number of lateral roots in wild-type and mutant plants from images taken using a linear robot camera. Primary root length and the number of lateral roots were measured using Image J (*33*) and SmartRoot software (*20*). The density of lateral roots was calculated dividing the number of lateral roots by the length of the principal root.

e. Computational and statistical analysis

For the lateral root density comparisons in the genotype/stress context, within each genotype/stress, we compared the effect of each microbial treatment (No bacteria (NB), Synthetic community (SynCom), Natural community) on lateral root density via the fitting of a linear model within each microbial treatment. The model had the following formula:

Lateral root density ~ Microbial treatment

Statistical significance across microbial treatments was determined via the emmeans function from the emmeans v.1.7.3, R package (*27*) and represented through the compact letter display via the cld function from the multcomp v.1.4-19, R package (*27*).

To compare the proportion of changes in lateral root density in wild-type Col-0 plants colonized by a 16-member bacterial synthetic community and a microbial natural community grown under two abiotic stresses (high salinity and low iron) to plants grown under full nutrient conditions (0.5x MS), we calculated for each stress condition the ratio between the average lateral root density in each bacterial treatment (SynCom and Natural community) and its respective non-inoculated control. Statistical significance between proportion of changes in lateral root density in response to bacterial treatment in each stress condition and the same value in the control condition (full nutrient) was determined via a Dunnet test implemented in the glht function of multcomp v. 1.4-19, R package (*27*) taking as input the fit of a linear model with the following formula:

Lateral root density ~ nutrient

Data Availability

RNA-Seq raw sequence data is available at the NCBI Gene Expression Omnibus under accession no. GSE210742. All data and code needed to reproduce all analyses can be

found at https://github.com/isaisg/rootbranchingmicro. All other data are present either in the main paper or the supplementary materials.

SI References

1. Banda, J., et al. Lateral Root Formation in Arabidopsis: A Well-Ordered LRexit. Trends Plant Sci. 24, 826–839 (2019).
2. Wachsman, G., et al. Cell wall remodeling and vesicle trafficking mediate the root clock in Arabidopsis. *Science (80-.).* **370**, 819–823 (2020).
3. Ursache, R., et al. GDSL-domain proteins have key roles in suberin polymerization and degradation. *Nat. plants*. **7**, 353–364 (2021).
4. Salas-González, I., et al. Coordination between microbiota and root endodermis supports plant mineral nutrient homeostasis. *Science*. **371** (2021)
5. Barberon, M., et al. Adaptation of Root Function by Nutrient-Induced Plasticity of Endodermal Differentiation. *Cell*. **164**, 447–459 (2016).
6. Manzano, C., et al. Auxin and Epigenetic Regulation of SKP2B, an F-Box That Represses Lateral Root Formation. *Plant Physiol.* **160**, 749 (2012).
7. Goh, T., Joi, S., Mimura, T., Fukaki, H. The establishment of asymmetry in Arabidopsis lateral root founder cells is regulated by LBD16/ASL18 and related LBD/ASL proteins. *Development*. **139**, 883–893 (2012).
8. Powers, S. K., et al. Nucleo-cytoplasmic Partitioning of ARF Proteins Controls Auxin Responses in Arabidopsis thaliana. *Mol. Cell*. **76**, 177-190.e5 (2019).
9. Chaiwanon, J., Wang, Z. Y. Spatiotemporal brassinosteroid signaling and antagonism with auxin pattern stem cell dynamics in Arabidopsis roots. *Curr. Biol.* **25**, 1031–1042 (2015).
10. Omelyanchuk, N. A., et al. Auxin regulates functional gene groups in a fold-change-specific manner in Arabidopsis thaliana roots. *Sci. Reports.* **7**, 1–11 (2017).

11. Mozgová, I., Muñoz-Viana, R., Hennig, L. PRC2 Represses Hormone-Induced Somatic Embryogenesis in Vegetative Tissue of Arabidopsis thaliana. *PLoS Genet.* **13**, 1006562 (2017).

12. Gala, H. P., et al. A single-cell view of the transcriptome during lateral root initiation in Arabidopsis thaliana. *Plant Cell*. **33**, 2197–2220 (2021).

13. Bhargava, A., et al. Identification of cytokinin-responsive genes using microarray meta-analysis and RNA-Seq in Arabidopsis. *Plant Physiol.* **162**, 272–294 (2013).

14. Castrillo, G., et al. Root microbiota drive direct integration of phosphate stress and immunity. *Nature*. **543**, 513–518 (2017).

15. Klepikova, A. V., Kasianov, A. S., Gerasimov, E. S., Logacheva, M. D., Penin, A. A. A high-resolution map of the Arabidopsis thaliana developmental transcriptome based on RNA-seq profiling. *Plant J.* **88**, 1058–1070 (2016).

16. Hou, S., et al. A microbiota–root–shoot circuit favours Arabidopsis growth over defence under suboptimal light. *Nat. Plants*. **7**, 1078 (2021).

17. Berens, M. L., et al. Balancing trade-offs between biotic and abiotic stress responses through leaf age-dependent variation in stress hormone cross-talk. *Proc. Natl. Acad. Sci. U. S. A.* **116**, 2364–2373 (2019).

18. Levy, A., et al. Genomic features of bacterial adaptation to plants. *Nat. Genet.* **50**, 138–150 (2017).

19. Bai, Y., et al. Functional overlap of the Arabidopsis leaf and root microbiota. *Nature*. **528**, 364–369 (2015).

20. Lobet, G., Pagès, L., Draye, X. A novel image-analysis toolbox enabling quantitative analysis of root system architecture. Plant Physiol. 157, 29–39 (2011).

21. Wu, M., Eisen, J. A. A simple, fast, and accurate method of phylogenomic inference. *Genome Biol.* **9** (2008), doi:10.1186/GB-2008-9-10-R151.

22. Katoh, K., Misawa, K., Kuma, K. I., Miyata, T. MAFFT: a novel method for rapid

multiple sequence alignment based on fast Fourier transform. *Nucleic Acids Res.* **30**, 3059–3066 (2002).

23. Capella-Gutiérrez, S., Silla-Martínez, J. M., Gabaldón, T. trimAl: a tool for automated alignment trimming in large-scale phylogenetic analyses. *Bioinformatics.* **25**, 1972–1973 (2009).
24. Price, M. N., Dehal, P. S., Arkin, A. P. FastTree 2--approximately maximum-likelihood trees for large alignments. *PLoS One*. **5** (2010).
25. Letunic, I., Bork, P. Interactive Tree Of Life (iTOL) v4: recent updates and new developments. *Nucleic Acids Res.* **47** (2019).
26. Revell, L. J. phytools: An R package for phylogenetic comparative biology (and other things). *Methods Ecol. Evol.* **3**, 217–223 (2012).
27. Oksanen, J. Vegan: Community Ecology Package. R package version 1.8-5 (2007).
28. Caspi, R., et al. The MetaCyc database of metabolic pathways and enzymes - a 2019 update. *Nucleic Acids Res.* **48**, D445–D453 (2020).
29. Chen, I. M. A., et al. IMG/M v.5.0: an integrated data management and comparative analysis system for microbial genomes and microbiomes. *Nucleic Acids Res.* **47**, D666–D677 (2019).
30. Finkel, O. M., et al. A single bacterial genus maintains root growth in a complex microbiome. *Nature*. **587**, 103–108 (2020).
31. Katoh, K., Rozewicki, J., and Yamada, K. MAFFT online service: multiple sequence alignment, interactive sequence choice and visualization. *Briefings in Bioinformatics* **20**:1160-1166 (2019).
32. Brunoud, G., et al. A novel sensor to map auxin response and distribution at high spatio-temporal resolution. *Nature*. **482**, 103–106 (2012).
33. Schindelin, J. et al. Fiji: an open-source platform for biological-image analysis. *Nat. Methods*. **9**, 676–682 (2012).

34. Lahner, B., et al. Genomic scale profiling of nutrient and trace elements in Arabidopsis thaliana. *Nat. Biotechnol.* **21**, 1215–1221 (2003).

35. Bric, J. M., Bostock, R. M., Silverstone, S. E. Rapid in situ assay for indoleacetic Acid production by bacteria immobilized on a nitrocellulose membrane. *Appl. Environ. Microbiol.* **57**, 535–538 (1991).

36. Gordon, S. A., Weber, R. P. COLORIMETRIC ESTIMATION OF INDOLEACETIC ACID. *Plant Physiol.* **26**, 192–195 (1951).

37. Band, L. R., et al. Systems Analysis of Auxin Transport in the Arabidopsis Root Apex. *Plant Cell*. **26**, 862 (2014).

38. He, W., et al. A Small-Molecule Screen Identifies l-Kynurenine as a Competitive Inhibitor of TAA1/TAR Activity in Ethylene-Directed Auxin Biosynthesis and Root Growth in Arabidopsis. *Plant Cell.* **23**, 3944 (2011).

39. Brumos, J., et al. Local Auxin Biosynthesis Is a Key Regulator of Plant Development. *Dev. Cell*. **47**, 306-318.e5 (2018).

40. Saltveit, M. E. Aminoethoxyvinylglycine (AVG) reduces ethylene and protein biosynthesis in excised discs of mature-green tomato pericarp tissue. *Postharvest Biol. Technol.* **35**, 183–190 (2005).

41. Bulens, I., Van de Poel, B., Hertog, M.L.A.T.M., De Proft, M.P., Geeraerd, A.H., Nicolai, B.M. Protocol: an updated integrated methodology for analysis of metabolites and enzyme activities of ethylene biosynthesis. *Plant Methods.* **7**, 17 (2011).

42. Logemann, J., Schell, J., Willmitzer, L. Improved method for the isolation of RNA from plant tissues. *Anal. Biochem.* **163**, 16–20 (1987).

43. Stepanova, A. N., Yun, J., Likhacheva, A. V., Alonso, J. M. Multilevel Interactions between Ethylene and Auxin in Arabidopsis Roots. *Plant Cell*. **19**, 2169 (2007).

44. Lavenus, J., et al. Lateral root development in Arabidopsis: fifty shades of auxin. *Trends Plant Sci.* **18**, 450–458 (2013).

45. Chen, S., Zhou, Y., Chen, Y., Gu, J. fastp: an ultra-fast all-in-one FASTQ preprocessor. *Bioinformatics*. **34**, i884–i890 (2018).

46. Patro, R., Duggal, G., Love, M. I., Irizarry, R. A., Kingsford, C. Salmon: fast and bias-aware quantification of transcript expression using dual-phase inference. *Nat. Methods*. **14**, 417 (2017).

47. Toufighi, K., Brady, S. M., Austin, R., Ly, E., Provart, N. J. The botany array resource: e-Northerns, expression angling, and promoter analyses. *Plant J.* **43**, 153–163 (2005).

48. Fukaki, H., Tameda, S., Masuda, H., Tasaka, M. Lateral root formation is blocked by a gain-of-function mutation in the SOLITARY-ROOT/IAA14 gene of Arabidopsis. *Plant J.* **29**, 153–168 (2002).

49. Okushima, Y., Fukaki, H., Onoda, M., Theologis, A., Tasaka, M. ARF7 and ARF19 regulate lateral root formation via direct activation of LBD/ASL genes in Arabidopsis. *Plant Cell*. **19**, 118–130 (2007).

50. Alonso J. M., et al. Five components of the ethylene-response pathway identified in a screen for weak ethylene-insensitive mutants in Arabidopsis. *Proc. Natl. Acad. Sci. U. S. A.* **100**, 2992–2997 (2003).

51. Zhao, X. C., Qu, X., Mathews, D. E., Schaller, G. E. Effect of ethylene pathway mutations upon expression of the ethylene receptor ETR1 from Arabidopsis. *Plant Physiol.* **130**, 1983–1991 (2002).

52. Méndez-Bravo, A. et al. CONSTITUTIVE TRIPLE RESPONSE1 and PIN2 act in a coordinate manner to support the indeterminate root growth and meristem cell proliferating activity in Arabidopsis seedlings. *Plant Sci.* **280**, 175–186 (2019).

53. Bürstenbinder, K., Rzewuski, G., Wirtz, M., Hell, R., Sauter, M. The role of methionine recycling for ethylene synthesis in Arabidopsis. *Plant J.* **49**, 238–249 (2007).

54. Sauter, M., Cornell, K. A., Beszteri, S., Rzewuski, G. Functional analysis of

methylthioribose kinase genes in plants. *Plant Physiol.* **136**, 4061–4071 (2004).

55. Chinchilla, D., et al. A flagellin-induced complex of the receptor FLS2 and BAK1 initiates plant defence. *Nature*. **448**, 497–500 (2007).
